# Rab11a Is Essential for the Development and Integrity of the Stereocilia and Kinocilia in the Mammalian Organ of Corti

**DOI:** 10.1523/ENEURO.0420-22.2023

**Published:** 2023-06-02

**Authors:** Lindsey Knapp, Haojie Sun, Yan-Mei Wang, Bin-Jun Chen, Xi Lin, Nan Gao, Ping Chen, Dongdong Ren

**Affiliations:** 1 Department of Cell Biology, Emory University, Atlanta, GA 30322; 2 Department of Otolaryngology-Head and Neck Surgery, Emory University School of Medicine, Atlanta, GA 30322; 3 Department of Otology and Skull Base Surgery, Eye and ENT Hospital of Fudan University, Shanghai 200031, China; 4 Department of Biological Sciences, Rutgers University, Newark, NJ 07102

**Keywords:** apical-basal protein targeting, intraflagellar transport, planar cell polarity, primary cilia, stereocilia, tip link

## Abstract

The cochlea hair cells transform mechanic sounds to neural signals with a remarkable sensitivity and resolution. This is achieved via the precisely sculpted mechanotransduction apparatus of the hair cells and the supporting structure of the cochlea. The shaping of the mechanotransduction apparatus, the staircased stereocilia bundles on the apical surface of the hair cells, requires an intricate regulatory network including planar cell polarity (PCP) and primary cilia genes in orienting stereocilia bundles and building molecular machinery of the apical protrusions. The mechanism linking these regulatory components is unknown. Here, we show that a small GTPase known for its role in protein trafficking, Rab11a, is required for ciliogenesis in hair cells during development in mice. In addition, in the absence of Rab11a, stereocilia bundles lost their cohesion and integrity, and mice are deaf. These data indicate an essential role of protein trafficking in the formation of hair cell mechanotransduction apparatus, implicating a role of Rab11a or protein trafficking in linking the cilia and polarity regulatory components with the molecular machinery in building the cohesive and precisely shaped stereocilia bundles.

## Significance Statement

Our research discovered for the first time that the small GTPase Rab11a is required for ciliogenesis, and for the precise patterning and cohesion of the stereocilia bundles in cochlear hair cells.

## Introduction

The auditory sensory organ, the organ of Corti, is housed within the cochlea on the flexible basilar membrane. It is responsible for transforming sound waves and vibrations received by the sensory hair cells to electrical signals that are relayed through the auditory neural circuit to the brain cortex for sound perception (Dallos, 1996; Hudspeth, 2000b). The auditory system is capable of processing sound over a remarkably wide frequency and volume range in an instant (Dallos, 1996; Hudspeth, 2000a).

The functional sensitivity, resolution, and adaptation of intensity and time of audition is supported by the unique physical features of mechanotransduction apparatuses and the cellular structure of the organ of Corti. The organ of Corti is composed of four rows of sensory hair cells of two distinct types, the inner hair cells (IHCs) and outer hair cells (OHCs). The inner hair cells (IHCs) are the primary cochlear transducers of sound while the afferent-innervated outer hair cells (OHCs) are endowed with motility, which is thought to allow for functional selectivity and sensitivity (Evans et al., 1991; Holley, 1996; Hudspeth, 1997; Y.C. Wu et al., 1999; Ricci et al., 2000; Ricci, 2003). Each hair cell is adorned with a V-shaped or U-shaped hair bundle consisting of actin-filled stereocilia and a single transit microtubule-based true cilium, the kinocilium. The stereocilia are graded in height, linked by interstereocilia and tip links, and patterned precisely with the tallest stereocilia at the vertex of the stereocilia bundle (Dallos, 1996; Hudspeth, 2000b). The kinocilium, which is a true cilium and primary cilium, emanate from the basal body near the vertex of the stereocilia bundle, and is present transiently during development (Sobkowicz et al., 1995). All the hair cells within the cochlea are oriented with their stereocilia bundles pointing to the periphery of the cochlear duct depicting the most distinctive form of planar cell polarity (PCP) in vertebrates (Rida and Chen, 2009). This uniform orientation is vital as the directional deflection of the stereociliary bundle gates the mechanoelectrical transducer (MET channel) located at the stereocilia (Tompkins et al., 2017). The staircase arrangement, the cohesion between individual stereocilia, and the uniform orientation of stereocilia bundles provide a structural foundation for mechanotransduction with high sensitivity and resolution (Kitamura et al., 1992; Alagramam et al., 2001; Bolz et al., 2001; Johnson et al., 2003; Zheng et al., 2005).

The molecular pathways that regulate the building and polarity of the stereocilia bundles are unveiled by genetic studies in human diseases and in animal models. Usher syndrome is a genetically diverse disease where mutations in at least 10 proteins affect hearing, vision, and balance (Cosgrove and Zallocchi, 2014; Zou et al., 2017). Mutations in the Usher complex proteins, which participate in building the polar structure of the stereocilia and kinocilium bundle on the apical surface of hair cell, cause dysmorphic stereocilia including bundles that are fragmented, altered in length and width, malformed kinocilia ultimately perturbing development and maintenance of the mechanotransduction apparatuses of the sensory hair cells. The polarity of each hair cells is regulated by the basal body and kinocilial genes (Ross et al., 2005; Jones et al., 2008), and the coordinated orientation of all hair cells, or PCP, is directed through asymmetrically localized membrane PCP proteins including Vangl2 and Frizzled3 at the junctions between the neighboring cells across the organ of Corti (Montcouquiol et al., 2003, 2006; Y. Wang et al., 2006). Moreover, cell polarity regulators classically involved in spindle orientation work with the PCP pathway to provide the blueprint for the apical surface of hair cells and outlines the contour of the stereocilia and the positioning of the kinocilium with promote orientation and development of the stereociliary bundles, with mInsc/Lgn/Ga_i_ laterally demarking the bare zone where the kinocilium is located and Par3/Par6/aPKC on the opposing medial side (Tarchini et al., 2013). These spindle regulators appear to be downstream of PCP proteins but upstream of kinocilia positioning as Ga_i_ localization is dependent on PCP protein localization but Ga_i_ and LGN direct kinocilia migration during development (Bhonker et al., 2016; Ezan et al., 2013; Tarchini et al., 2013).

To achieve cellular and/or tissue polarity, proteins have to be targeted to specific cellular locations to signal the directionality in the cells and across the tissue, and to build the specific polarity structure that is unique for each cell. Our recent study has identified a role for endocytic pathways (Lim et al., 2011) in trafficking membrane PCP protein Vangl2 (Tower-Gilchrist et al., 2019). However, the protein targeting mechanisms in building the polarized structure of the bundle of stereocilia and the kinocilium remains unknown.

Studies of protein trafficking have implicated roles for Rab11 in protein targeting in the apical domain of epithelial cells and in ciliogenesis (Welz et al., 2014). Rab11 delivers biosynthetic cargo to the apical surface through the trans Golgi network and redistribution of proteins through vesicles of the recycling endosome (Urbé et al., 1993; Ullrich et al., 1996; Casanova et al., 1999; Lapierre et al., 2001; Hales et al., 2002). During ciliogenesis in cultured cells, Rab11 is required for Rabin8 targeting to the basal body, which activates Rab8 membrane extension of the cilia and protein vesicle docking in conjunction with the BBSome, allowing protein entry and targeting to the cilia tip via intraflagellar transport proteins (Nachury et al., 2007; Jin et al., 2010; Knödler et al., 2010; J. Wang et al., 2012; Westlake et al., 2011).

Conditional knock-out (CKO) studies of members of the Rab11 family have revealed a specific role for Rab11a in apical protein trafficking, including Ezrin and Syntaxin3 and the formation of microvilli (Sobajima et al., 2014; Yu et al., 2014; Knowles et al., 2015). Rab11a was initially described as a protein of the recycling endosome and trans Golgi network responsible for apical protein trafficking in epithelial cells (Urbé et al., 1993; Ullrich et al., 1996). Additionally, Rab11a was found to be a major player in the recycling endosome allowing membrane proteins to be endocytosed, sorted, and redistributed to the plasma membrane allowing for trancytosis of proteins or a final distribution of proteins (Ducharme et al., 2007). Several researchers have implicated the Rab11 family in planar cell polarity. *In vitro*, Rab11a marked recycling endosomes carried Celsr1 when PCP proteins were internalized and redistributed during cell divisions (Devenport et al., 2011). Additionally, Rab11 family has been implicated in multiple processes in *Xenopus* embryogenesis and trafficking Vangl2, a major component of the PCP pathway (Ohyama and Groves, 2004; Kim et al., 2012; Ossipova et al., 2014, 2015). Recent research revealed that Rab11a affected the distribution of polarity proteins (Vangl2, Prickle-2) and development of cilia in the mouse vestibular organs (Chen et al., 2021). However, whether Rab11a protein participates in protein targeting in the apical domain of hair cells for the polarized bundle of stereocilia and kinocilium and planar cell polarity in the cochlear remains unknown.

In this study, we investigated a potential role for Rab11a in apical structures of the hair cells in the cochlea. We found that Rab11a is required for the formation of primary cilia in mouse embryonic fibroblasts (MEFs) and the kinocilium in hair cells, and for development and integrity of the stereocilia bundle in hair cells.

## Materials and Methods

### Mouse strains and animal care

Animal care was compliant NIH guidelines and was approved by Emory University institutional animal care and use committee (IACUC). *Rab11a* conditional knock-out alleles, Vangl2-Looptail mice (The Jackson Laboratory Jax stock #000220), and *IFT88* conditional knock-out alleles were described previously (Kibar et al., 2001; Haycraft et al., 2007; Yu et al., 2014). *Rab11a* and *IFT88* conditional alleles were inactivated via Cre-recombinase driven by Pax2Cre (Ohyama and Groves, 2004).

#### RT-PCR

Cochlear epithelia were dissected from embryonic day (E)14.5, E16.5, and postnatal day (P)0 wild-type (WT) mice and stored at −80°Celcius until RNA isolation. RNA was isolated using RNeasy Miki kit (QIAGEN) homogenizing with QIAshredder homogenizers (QIAGEN) and DNase digestion with DNase 1 recombinant, RNase-free (Roche) per manufacturer’s instructions. Cochlear cDNA was made form isolated RNA using M-MLV Reverse Transcriptase (Invitrogen) with oligoDT and random primers per manufacturer’s instructions. Rab11a primers were (forward) 5′-CCAGGTTGATGGGAAAACAATA-3′ and (reverse) 5′-AGACATGTCATTTTCACGTCT-3′ and Rab11b primers were (forward) 5′-ACGCTTCACCAGAAACGAATTC-3′ and (reverse) 5′-CAGGGGACTCATCGTGGGC-3′.

### MEF cell culture experiment

Production of wild-type and Rab11a null mouse embryonic fibroblasts (MEFs) were described previously (gift from Nan Gao, Rutgers University, Newark, NJ; Yu et al., 2014). MEFs were serum starved for at least 13 h before staining to induce ciliation. Rescue of Rab11a-null MEFs was performed by transfecting DsRed-Rab11a plasmids using Lipofectamine 2000 (Invirtogen) and standard protocols. Cells on coverslips were fixed with 4% paraformaldehyde in PBS for 15 min, permeabilized in 0.1% Triton X-100 in PBS (PBS-T) for 10 min, blocked with 10% donkey in PBS-T serum for 1 h, and incubated with primary and secondary antibodies. Cells were scored for ciliation and analyzed as described below (Cilia Quantification).

### Inner ear dissection, immunostaining, and imaging

Standard procedures were used for the dissection and immunostaining of inner ears. Briefly, temporal bones from E18.5 to P2 were harvested and fixed in 4% paraformaldehyde in PBS for 2 h at room temperature, 2 h on ice, or overnight at 4°C. Temporal bones were washed with PBS and stored in PBS at 4°C until microdissection.

The organ of Corti was microdissected and blocked in 10% donkey serum in 0.1% Triton X-100 in PBS (10% DS in PBS-T) for 1 h at room temperature. Organ of Corti tissue was incubated in primary antibody in 5% DS in PBS-T overnight at 4°C. After three 5-min washes in PBS-T, tissue was incubated in secondary antibody and/or phalloidin in 5% donkey serum and PBS-T for 2 h at room temperature. Tissues were again washed three times for 5 min each in PBS-T. Samples were mounted in Fluoromount-G (SouthernBiotech, #0100-01) with 1.5 coverslips and sealed.

The following primary antibodies were used: Rab11a (Cell Signaling Technology #2413, 1:200), γ-tubulin (Sigma, #T6557, 1:200; Lee et al., 2019), Arl13b (Tamara Caspary, Emory University, Atlanta, GA; 1:1500), Vangl2 (R&D Systems #AF4815, 1:200; Luo et al., 2017), Fz3 (Gift from Jeremy Nathans, Johns Hopkins University, Baltimore, MD, 1:500; Y. Wang et al., 2006), LGN (gift from Fumio Matsuzaki, RIKEN, 1:200; Konno et al., 2008), β-Spectrin (BD Transduction Laboratories #612562, 1:200), MyosinVIIa (Proteus Bioscience Inc. #25-6790, 1:200; Prajapati-DiNubila et al., 2019), Radixin (Abcam #ab52495, 1:100; Mu et al., 2018), and E-Cadherin (Invitrogen #13-1700, 1:200; Benedicto et al., 2017).

Confocal Images were obtained using Olympus FV1000/TIRF or Zeiss LSM510 confocal microscopes. Image analysis including production of orthogonal views, Z-projections, and figures was completed in ImageJ (NIH) and Adobe Photoshop.

### Scanning electron microscopy

Mouse organ of Corti samples were fixed in 3 mm calcium chloride, 2.5% glutaraldehyde in 0.1 m cacodylate and allowed to fix overnight. Samples were then rinsed with 0.1 m cacodylate buffer followed by postfixed in 1% osmium tetroxide in 0.1 m cacodylate for 1 h and rinsed in deionized water. The samples were dehydrated through an ethanol series and then placed in 100% dry ethanol. The samples were placed into labeled microprous specimen capsules and loaded into the sample boat of a chilled Polaron E3000 critical point drying unit. The unit was sealed and filled with liquid CO_2_ under pressure. The CO_2_ was allowed to gently wash through the chamber and exchange for the ethanol in the tissue. When the exchange was complete, the CO_2_ was brought to its critical point of 1073 psi and 31°C and allowed to gently bleed away.

The dry samples were mounted on labeled SEM stubs and then coated using a Denton Vacuum Desk II sputter coater with a Gold/Palladium target. The samples were imaged at 10 kV using the lower stage of a Topcon DS130 field emission scanning electron microscope (SEM) and images collected using a Quartz PCI digital image collection system.

### Phenotypic and statistical analysis

#### Stereocilia morphology

Stereocilia morphology of IHCs and OHCs was counted and scored based on the shape of the stereocilia bundles as follows: normal stereocilia have an inversed U-shaped or V-shaped bundles, split stereocilia have two or more groupings of stereocilia which have split apart from one another, circular stereocilia have stereocilia that are no longer in the U or V shape but now in the shape of an “O,” and flat stereocilia are connected in a line but there is no vertex. For each genotype, three independent cochleae were counted and quantified noting the region and hair cell type. Statistical tests were run using a *t* test for samples where two genotypes were being compared, a one-way ANOVA to compare regions in a single genotype, and a two-way ANOVA to compare between regions between genotypes. *p*-values ≤0.05 were considered significant.

#### Stereocilia orientation

To determine stereocilia orientation, a line was drawn across the planar axis and another line was drawn to bisect the middle of the V-shaped stereocilia bundle to create an angle. In the absence of properly formed stereocilia the actin devoid fonticulus, typically at the vertex of the V-shaped stereocilia bundles was used to determine the hair cell’s orientation. This angle was compared with the angle formed by the planar axis to the medial-lateral axis of the cell (90°). Only the apex of the hair cell was counted as no apparent deviation in orientation was seen in the other regions. The hair cell row was recorded and at least 75 cells form each hair cell row was counted for each of the three samples per genotype. The most lateral row of hair cells of the apex of the cochlea was used to statistical analysis in Oriana3. A χ^2^ test was used to compare the proportion of cells deviating from 30° from the normal angle of 90° between genotypes.

#### Cilia quantification

Presence of a cilia marked by Arl13b staining was recorded and separated by IHC row and OHC row. A minimum of 300 IHCs and 1200 OHCs were counted per each region of each genotype. Three adjacent fields of view in each region (basal/middle/apex) and five individual animal samples per each condition were included in quantification. Statistical tests were run as described above for the stereocilia phenotype.

#### General statistical analysis and software

Software for statistical analysis was used from www.graphpad.com/quickcalcs, and a *p*-value of 0.05 or lower was considered significant unless otherwise noted.

### Auditory brainstem response (ABR) measurement

ABR measurements were conducted within a sound attenuating booth (Shanghai Shino Acoustic Equipment Co, Ltd). Mice were anaesthetized with chloral hydrate (480 mg/kg, i.p.), and then were placed onto a small animal heating pad to maintain body temperature (Automatic thermostation, BORO Zoo Co, Ltd). Subdermal needle electrodes (Rochester Electro-Medical) were placed at the vertex (active, noninverting), the left infra-auricular mastoid region (reference, inverting), and the right infra-auricular mastoid region(ground). The acoustic stimuli for ABR were produced by software SigGenRZ and the responses recorded using a TDT system controlled by BioSigRZ, digital signal processing software (Tucker-Davis Technologies). Differentially recorded scalp potentials were bandpass filtered between 0.3 and 3 kHz over a 20-ms epoch. A total of 400 trials were averaged for each stimulus condition. ABRs were elicited with digitally generated (SigGenRZ, TDT) pure tone pips presented free field via a speaker (TDT, Part MF1 2020) positioned 10 cm from the vertex. Symmetrically shaped tone bursts were 3 ms long (1 ms raised cosine on/off ramps and 1-ms plateau) and were delivered at a rate of 20 per second. Stimuli were presented at frequencies at 8, 16, 24, and 32 kHz and in 5-dB decrements of sound intensity from 90 to 20 dB SPL. The ABR threshold was defined as the lowest intensity (with 5-dB resolution) capable of evoking a reproducible, visually detectable response with S/N ratio of ∼1.5.

## Results

### Rab11a is localized adjacent to the basal body in cochlear hair cells

**Figure 1.**
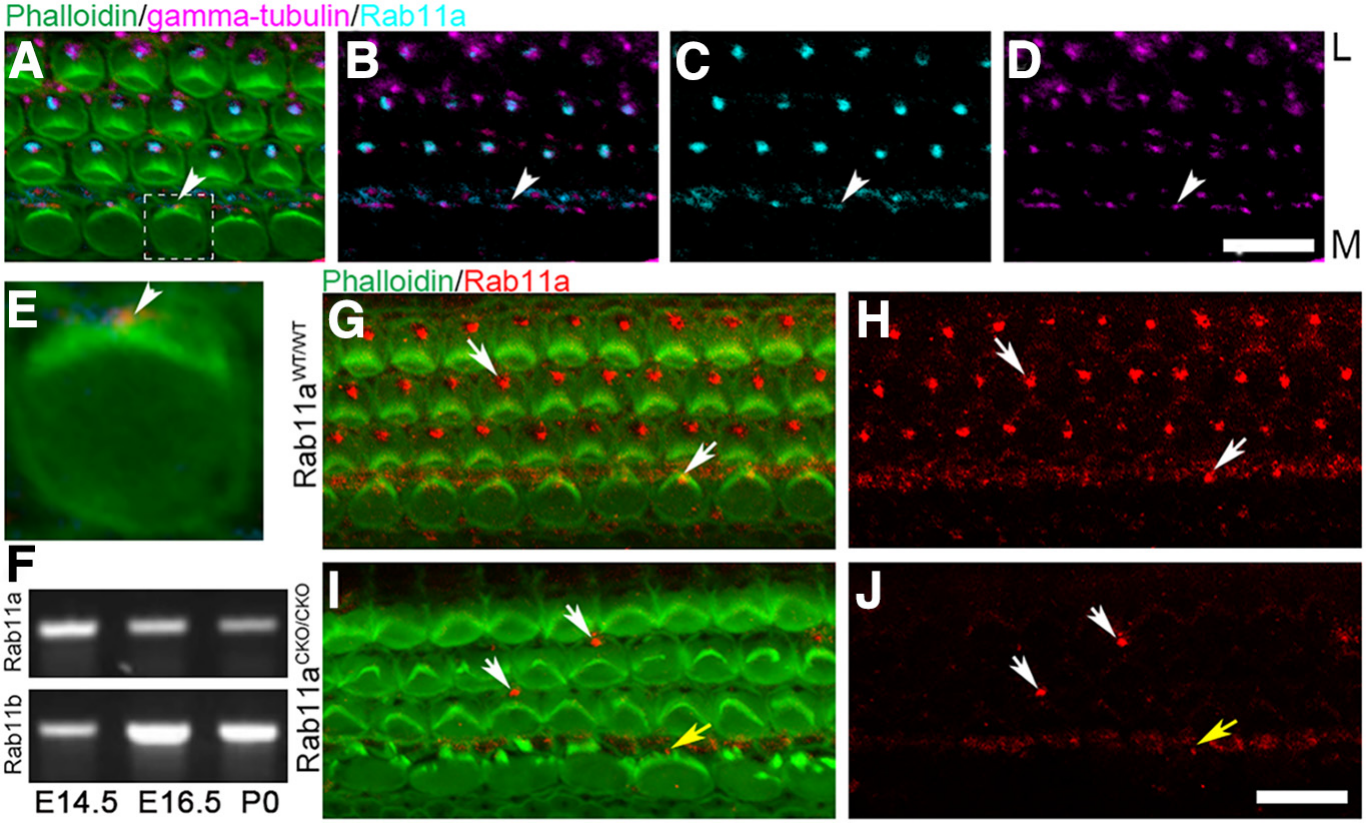
Rab11a is expressed in the organ of Corti and localized adjacent to the basal body of sensory hair cells. ***A–E***, Cochlea from a wild-type postnatal day (P)0 mice stained with phalloidin (actin/stereocilia, green), γ-tubulin (basal body, magenta), and Rab11a (cyan). Hair bundle is composed of actin-rich stereocilia arranged in a V shape with the basal body at the vertex of the V-shaped stereocilia bundle. The first row of hair cells at the medial (M) or center of the cochlea duct are inner hair cells, while the three rows of hair cells lateral (L) are the outer hair cells. An inner hair cell marked by a dashed box in ***A*** (***E***). The basal body of the inner hair cell is indicated by an arrowhead. M: medial, L: lateral. Scale bar: 10 μm. ***F***, RT-PCR of Rab11a and Rab11b from cDNA isolated from cochlea of embryonic day (E)14.5, E16.5, and P0 mice. Rab11a and Rab11b specific primers were used as described in Materials and Methods. ***G–J***, Cochleae from P2 mice containing Rab11a floxed allele (Rab11afl/fl) or P2 Rab11afl/fl; Pax2-Cre (***I***, ***J***) mice stained with Phalloidin (actin, green) and Rab11a (red). White arrows indicate Rab11a localization near the vertex of the V-shaped stereocilia bundle in the cochlea with the floxed alleles but no Cre activation (wild type; ***G***, ***H***). In mice where Rab11a was conditionally knocked out by Pax2Cre (Rab11aCKO/CKO), Rab11a protein staining is mostly not detectable (***I***, ***J***). The hair cells with remaining Rab11a protein staining near the vertex of the outer hair cell stereocilia are indicated by white arrows. The yellow arrow marks an inner hair cell with an intact stereocilia bundle. Scale bar: 10 μm.

To start exploring the role of Rab11a in the cochlea, we investigated the localization of Rab11a in the cochlea. Stereocilia, actin rich modified microvilli were visualized with phalloidin while basal bodies, from which the kinocilia is projected, were stained using an antibody against γ-tubulin (Fig. 1*A–E*). Rab11a is highly homologous with Rab11b (Bock et al., 2001; Pereira-Leal and Seabra, 2001), and both Rab11a and Rab11b mRNA were detected in cochlear epithelia throughout development (Fig. 1*F*).Using a Rab11a-specific antibody that was raised against an epitope specific to Rab11a (Cell Signaling Technology #2413), we found Rab11a localized near the basal body at the vertex of the V-shaped and U-shaped stereocilia bundles in sensory hair cells (HCs; Fig. 1*A–E*). This localization is consistent with reports of Rab11 in the basal body in cultured cells and the cochlea (Knödler et al., 2010; Kirjavainen et al., 2015).

The specificity of the Rab11a antibody in the cochlea was confirmed by examination of the Rab11a staining in Rab11a conditional knock-out cochleae. We bred *Pax2^Cre^* mice (Ohyama and Groves, 2004) with mice carrying a floxed allele of *Rab11a* (Yu et al., 2014) to generate Rab11a inner ear-conditional knock-out mice (Rab11a CKO). The Pax2 Cre mRNA starts to be expressed in otocyst, kidney, and midbrain–hindbrain boundary at E9.5 (Ohyama and Groves, 2004). Rab11a signal was almost gone in Rab11a CKO cochlea confirmed the specificity of the antibody and the efficiency of the Rab11a conditional knock-out (Fig. 1*G–J*).

In summary, Rab11a was localized to the vicinity of the basal body in the cochlear HCs and its expression is mostly undetectable in Rab11a CKO mice.

### Inactivation of Rab11a leads to defects in stereocilia bundles of HCs with varying degrees along the longitudinal axes and between IHC and OHC of the cochlea

**Figure 2.**
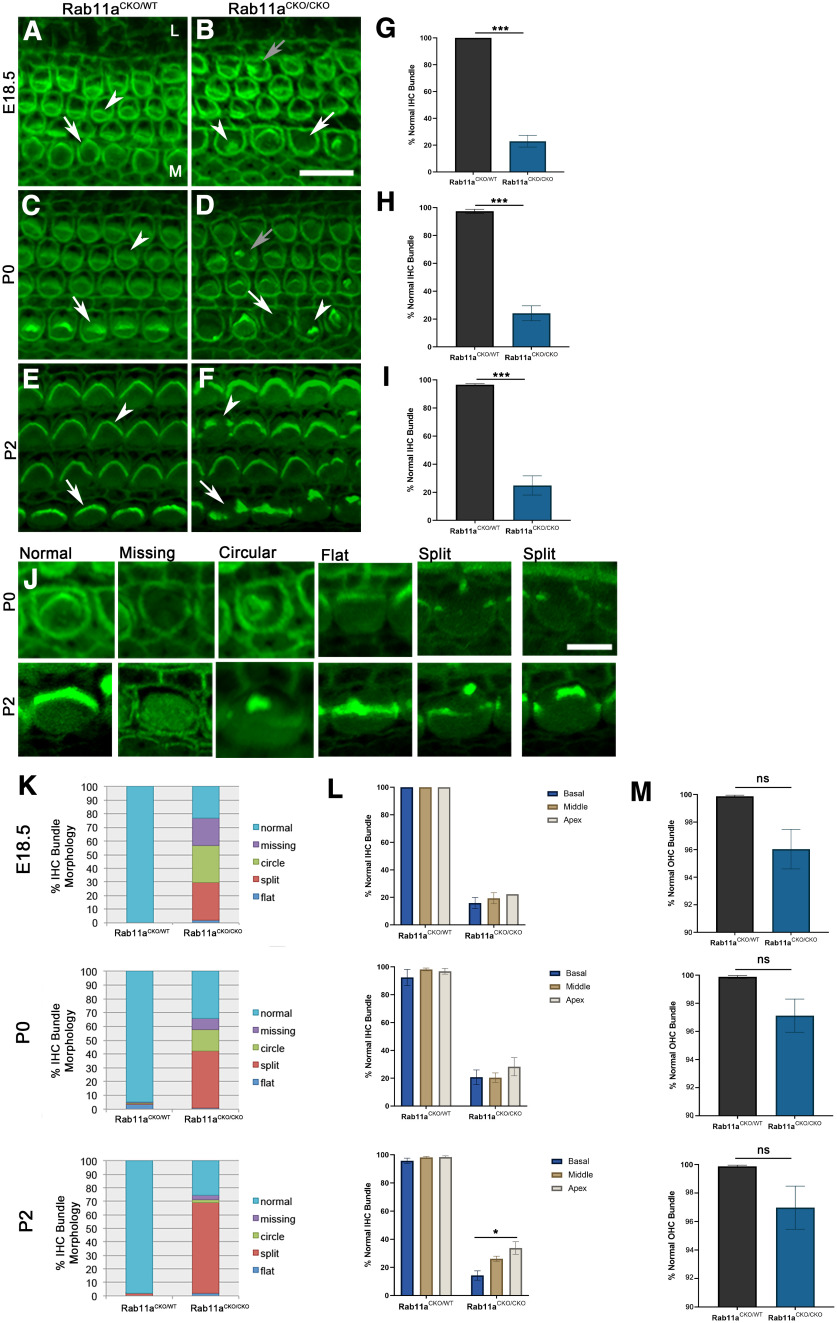
Depletion of Rab11a leads to abnormal development of hair bundles in cochlear sensory hair cells. ***A–F***, Cochlea from Rab11a heterozygous conditional knock-out (Rab11aCKO/WT; ***A***, ***C***, ***E***) and Rab11a homozygous conditional knock-out (Rab11aCKO/CKO) mice (***B***, ***D***, ***F***) at E18.5 (***A***, ***B***), P0 (***C***, ***D***), or P2 (***E***, ***F***) were stained with phalloidin (actin, green) to visualize the actin-filled stereocilia bundles. In Rab11aCKO/WT cochlear hair cells, stereocilia are patterned into a V-shaped hair bundle at the apical surface of both inner (arrow) and outer (arrowhead) hair cells (***A***, ***C***, ***E***). In Rab11aCKO/CKO cochlea, the inner and outer hair cells bundles are malformed most prominently in inner hair cells (***B***, ***D***, ***F***). In Rab11aCKO/CKO cochlea, there are inner hair cells with missing stereocilia (arrow), and circular or clustered stereocilia bundle (arrowhead) at E18.5 (***B***). In the outer hair cells, the stereocilia bundles are mostly normal while few malformed stereocilia bundles were observed (***B***, gray arrow). At P0 (***D***), abnormal hair bundle formation includes missing (arrow) or circular/clustered (arrowhead) in the inner hair cells and split in the outer hair cells (grew arrow). By P2 (***F***), the most frequently observed malformation of stereocilia are split hair bundles in the inner hair cells (arrow) and outer hair cells (arrowhead). Scale bar: 10 μm. M: medial, L: lateral. ***G–I***, Aberrant stereocilia bundle morphology of inner hair cell (IHC) was quantified and graphed as a percent of the total number of IHCs. Morphology comparison between heterozygous and homozygous Rab11a knock-out cochleae at E18.5 (***G***), P0 (***H***), and P2 (***I***) showed a significant reduction in normal stereocilia bundle morphology in homozygous Rab11a CKO cochleae. Data from three animals at each stage for each genotype were included and averaged with error bars representing SEM *t* test was conducted to determine the *p*-value. ***J***, Example of normal and abnormal (missing, circular or cluster, flat, and split) stereocilia phenotypes at P0 and P2. Scale bar: 5 μm. ***K***, Inner hair cell (IHC) stereocilia morphology phenotypes (normal, missing, circular, flat, or split) were counted for Rab11a heterozygous (HET) and homozygous (HOM) conditional knock-out at E18.5, P0, and P2. Percentages of each phenotype was graphed. ***L***, Normal IHC stereocilia bundle morphology was quantified for each region of the cochleae along the longitudinal base-apex axis of the cochlear duct. Unlike most other developmental phenotypes that often show more prominent phenotypes toward the apex of the cochlear duct, the stereocilia bundle phenotype in Rab11aCKO/CKO is strongest at the base of the cochleae. ***M***, Quantification of stereocilia morphology in outer hair cells (OHC) showed a nonsignificant reduction in normal stereocilia. Plotted with average and SEM of three samples for each genotype at each stage. *p*-values equal 0.0556 (E18.5), 0.0861 (P0), and 0.1274 (P2). *p < 0.05, **p < 0.001, ***p < 0.001. ns, not significant. Rab11a CKO was generated by crossing floxed allele of Rab11a with Pax2Cre mice. A minimum of 300 IHCs and 1200 OHCs were counted per each region of each genotype. Three adjacent fields of view in each region (basal/middle/apex) and five individual animal samples per each condition were included in quantification.

During cochlear development, HC differentiation and stereocilia bundle morphology maturation occur in a graded fashion from the mid-base to the apex along the longitudinal axis of the cochlear duct and from inner to outer hair cells along the mediolateral axis of the cochlear duct (Montcouquiol and Kelley, 2003). Each cochlear hair cell has precisely patterned hair bundles consisting of a staircase of stereocilia arranged in a V-shaped or U-shaped and a kinocilia near the vertex of the V-shaped or U-shaped stereocilia. The significant reduction of Rab11a expression in the cochlear HCs of mice with Rab11a CKO allowed us to analyze the role of Rab11a in the cochlea (Fig. 2).

At the end of gestation (E18.5), hair bundles are recognizable in all hair cells of control heterozygous littermates (Fig. 2*A*). However, missing or deformed stereocilia bundles in the Rab11a CKO at this time point were observed in the HCs (Fig. 2*B*). The IHCs were more influenced than OHCs. At birth (P0) and postnatal day (P)2 as the hair bundles achieve their distinct matured morphology, the stereocilia abnormality in the IHCs remains with a similar deformation of stereocilia bundles. But in the OHCs, slightly disorganized stereocilia bundles with less severity could be recognized (Fig. 2*C–F*).

We further quantified the stereocilia abnormality phenotype in both the IHCs (Fig. 2*G–I*) and OHCs (Fig. 2*K*), and classified the various abnormalities as missing bundles, circular bundle, flat bundle, and split or fragmented bundles (Fig. 2*J*,*L*). In the IHCs, the stereocilia bundle abnormality manifests as missing or circular in 47.7% at E18.5, missing or circular in 23.6% and fragmented in 41.1% at P0, and fragmented in 66.9% at P2 (Fig. 2*G–J*,*L*). In contrast, there were no statistical significance identified in OHCs. In conclusion, loss of Rab11a causes significant stereocilia bundle abnormalities in stereocilia bundles of the IHCs at E18.5, P0, and P2 (Fig. 2*G–I*).

To understand the stereocilia phenotype along the longitudinal axis in the Rab11a CKO cochlea, we analyzed the degree of stereocilia bundle morphology deficit. In control cochlea, stereocilia bundles are V-shaped or U-shaped with nearly 100% normal stereocilia bundle morphology along the longitudinal axis (Fig. 2*M*). In the Rab11a CKO, at E18.5, there is very little difference in the percent of normal bundle morphology from the apex to the base of the cochlea while the overall percentage of normal bundle morphology is significantly lower than that of the control cochlea (Fig. 2*L*). However, as development continues postnatally and achieves more distinct morphology by P2, the percentage of normal hair bundles in mutants is markedly different from that of controls, and the difference among sections along the longitudinal axis is statistically significant (Fig. 2*M*). Rab11a knock-out in the cochlea produces a stereocilia bundle phenotype that is more prominent in the more mature cells in the base of the cochlear duct (which indicates an obvious phenotype) and inner hair cells compared with the less mature apex and outer hair cells.

### Rab11a is required for hearing and its absence leads to hair cell degeneration in adult mice

**Figure 3.**
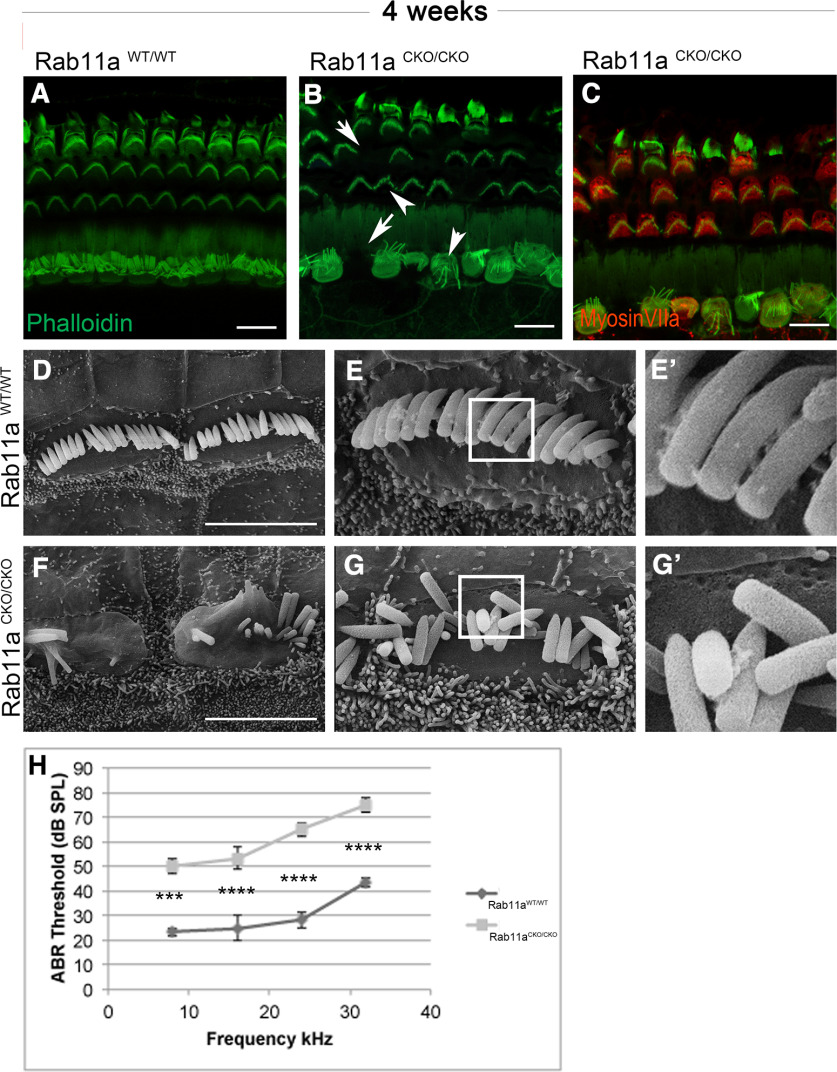
The absence of Rab11a causes hearing loss and hair cell degeneration. ***A–C***, Cochlea from four-week-old wild-type (***A***) and Rab11a CKO (***B***, ***C***) mice were stained with phalloidin (green) to reveal stereocilia. In wild-type, outer hair cells have neat V-shaped stereocilia bundles and inner hair cells with a characteristic U-shaped bundle with longer stereocilia (***A***). Rab11a CKO cochlea have missing hair cells (arrows) and stereocilia that are less organized and fragmented (arrowheads) in both inner and outer hair cell rows. Rab11a CKO cochlea were stained with MyosinVIIa antibody to visualize hair cells (***C***). Scale bar: 10 μm (***D–G***) Scanning electron micrographs (SEM) of IHCs from four-week-old wild-type (***D***, ***E***) and Rab11a CKO (***F***, ***G***). The cilia junction of IHCs were shown in zoomed graphs in right two columns (***E’***, ***G’***). Scale bar: 10 μm. (***H***) Four-week-old control (WT) and Rab11a CKO (HOM) mice were subject to auditory brainstem response (ABR) threshold test. A two-way ANOVA with *post hoc* Sidak’s multiple comparison test suggests significant change decrease in ABR threshold was observed in Rab11a CKO mice indicating significant hearing loss. ***p < 0.001, ****p < 0.0001.

In order to determine the long-term effects of Rab11a loss of the cochlea, we analyzed four-week-old mice for hearing function and their cochlea to determine the abnormalities of important hair cell structure (stereocilia bundle arrangement and interconnection). Adult cochlea from the mice were dissected and stained with phalloidin. Wild-type mouse cochlea have OHCs with V-shaped stereocilia bundles and IHCs with U-shaped bundles and longer stereocilia (Fig. 3*A*). In Rab11a CKO cochlea, loss of stereocilia could be observed by the lack of phalloidin staining indicating a failure in stereocilia maintenance (Fig. 3*B*). Additionally, stereocilia appear more fragmented in both the IHC and OHC row with IHC stereocilia bundles also appearing severely less organized (Fig. 3*B*). Staining of Rab11a CKO cochlea revealed that hair cells without phalloidin signal were also not labeled by MyosinVIIa (Fig. 3*C*), indicating that hair cells without stereocilia may be lost by four weeks of age. The stereocilia of IHC hair bundles of Rab11a mutants were observed under scanning electronic microscope in adults and showed a disorganized phenotype (Fig. 3*D–G*). Intact interstereociliary connectors between IHC stereocilia bundles of WT were observed, while these links were lost in IHC of Rab11a CKO cochlea (Fig. 3*E’*,*G’*). Auditory brainstem response (ABR) threshold tests performed on these mice showed normal hearing range for control animals while thresholds were significantly higher for Rab11a CKO mice for all frequencies tested indicating profound hearing loss (Fig. 3*H*). These data indicate that Rab11a is responsible for the development of hearing.

### Protein partitioning along the PCP and apical-basal axes of the organ of Corti is not apparently affected by Rab11a CKO

Rab11a has been found to affect PCP processes such as gastrulation and neural tube closure in *Xenopus* during embryogenesis (Kim et al., 2012; Ossipova et al., 2014, 2015). Likewise, *in vitro* data suggest that Rab11 is involved in PCP protein trafficking (Devenport et al., 2011) and Rab11 participates in trafficking Vangl2, a major component of the PCP pathway (Kim et al., 2012; Ossipova et al., 2014, 2015).

**Figure 4.**
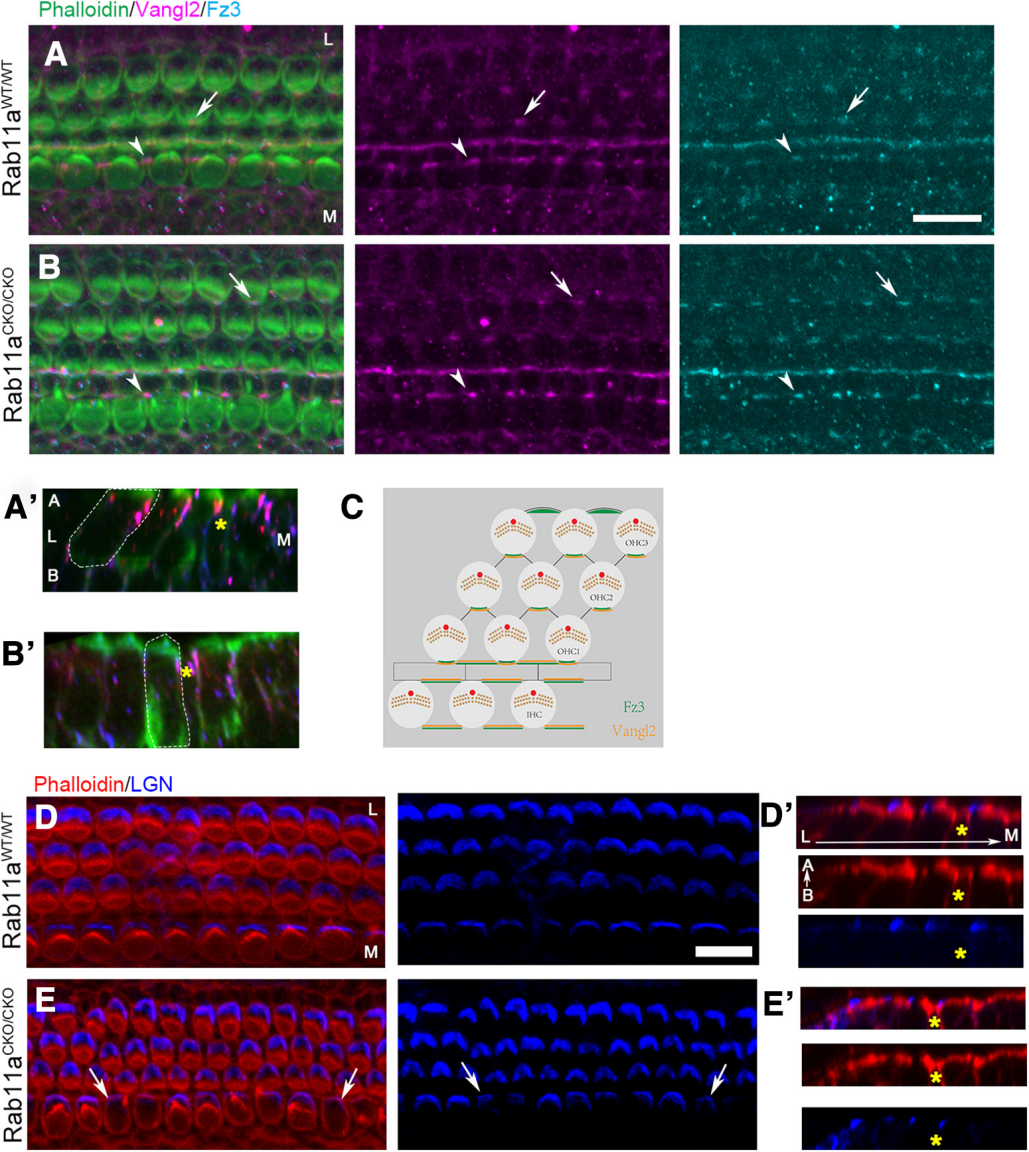
Apical protein partitioning along the planar cell polarity axis is not altered in Rab11a mutants. ***A–C***, Wild-type (***A***) and Rab11a homozygous conditional knock-out (***B***) cochlea at P0 were stained with phalloidin (green), Vangl2 (magenta), and Frizzled3 (cyan). In both the wild-type (***A***) and Rab11a knock-out (***B***) partitioning of planar cell polarity proteins Vangl2 and Frizzled3 are localized to the medial edge of hair cells where they contact supporting cells in the outer hair cells (arrow) and on the lateral sides of the supporting cells intercalated with the inner hair cells (arrowhead). Orthogonal view of wild-type (***A’***) and Rab11a knock-out (***B’***) have Vangl2 and Frizzled localized to the apical part of the hair cells. A schematic diagram the localization of Fz3 and Vangl2 was shown (***C***). ***D***, ***E***, Wild-type (***D***) and Rab11a homozygous conditional knock-out (Rab11aCKO/CKO; ***E***) cochlea at P0 was stained with phalloidin (stereocilia, red) and LGN (bare zone, blue). In the wild-type cochlea, LGN marks the bare zone lateral to the stereocilia. In the Rab11a homozygous conditional knock-out cochlea despite the loss of IHC stereocilia and stereocilia cohesion, LGN still forms on the lateral edge of the hair cell (arrow). Orthogonal views show that LGN retains its localization at the apical surface of the hair cells in both wild-type (***D’***) and homozygous knock-out (***E’***) hair cells. A yellow asterisk marks the pillar supporting cells between the inner and outer hair cell rows. Scale bars: 10 μm. L: lateral, M: medial, A: apical, B: basal.

First, we looked at localization of stereocilia patterning proteins in the PCP pathway. The PCP pathway directs the precise orientation of kinocilia and stereocilia orientation in HCs (Rida and Chen, 2009). PCP proteins such as Vangl2 and Frizzled3 are localized asymmetrically in hair cells and supporting cells along the mediolateral axis at the junction at the medial edge of hair cells and the lateral edge of supporting cells (Fig. 4*C*; Montcouquiol et al., 2006). Similarly, in Rab11a CKO cochlea, the asymmetrical localization of Vangl2 and Frizzled3 along the mediolateral axis was retained with no apparent differences from control cochlea (Fig. 4*A*,*B*). These data suggest that Rab11a is not necessary for the proper asymmetric mediolateral localization of Vangl2 and Frizzled3.

Second, we analyzed the localization of stereocilia patterning proteins that are thought to mark the localization of microvilli extension. Proteins LGN and Gαi are localized lateral to the stereocilia in the hair cells in the bare zone where microvilli retract during stereocilia elongation (Tarchini et al., 2013). aPKC, on the other hand, is localized medially to the stereocilia in the area where microvilli remain after stereocilia elongation. Since the localization of these proteins precede stereocilia formation, they have been termed the “blueprint” for stereocilia patterning. Additionally, mutations or loss of function of these proteins perturbs stereocilia patterning. We analyzed LGN localization in wild-type and Rab11a CKO cochlea to determine whether the stereocilia phenotype could be explained by mislocalization or loss of these stereocilia-patterning proteins (Fig. 4*D*,*E*). In controlled cochlea, LGN is localized to the region lateral to the stereocilia (Fig. 4*D*). Surprisingly, LGN was expressed in the Rab11a CKO cochlea, even in IHCs that completely lacked stereocilia (Fig. 4*E*). These data suggest that the stereocilia defect we observe is downstream of the LGN/molecular blueprint-patterning pathway.

Rab11 family has specifically been implicated in targeting of membrane proteins to the apex of the cell opposed to the basolateral surface. Since we did not see difference in protein localization across the planar axis of the cochlea, we decided to look at the apical-basal cell axis of the cochlea. Using orthogonal view, we were able to see the stereocilia, cuticular plate, and cell junction with phalloidin staining (Fig. 4*A’*,*B’*,*D’*,*E’*). LGN is localized to the lateral edge of each hair cell and is constricted to the most apical surface marked by the actin of the cuticular plate in both control and Rab11a CKO cochlea (Fig. 4*D’*,*E’*). The PCP proteins were also localized at the apical half of hair cells in controls but on the medial edge in both genotypes (Fig. 4*A’*,*B’*). These data suggest that Rab11a is not affecting apical localization of stereocilia patterning proteins in the cochlear HCs.

**Figure 5.**
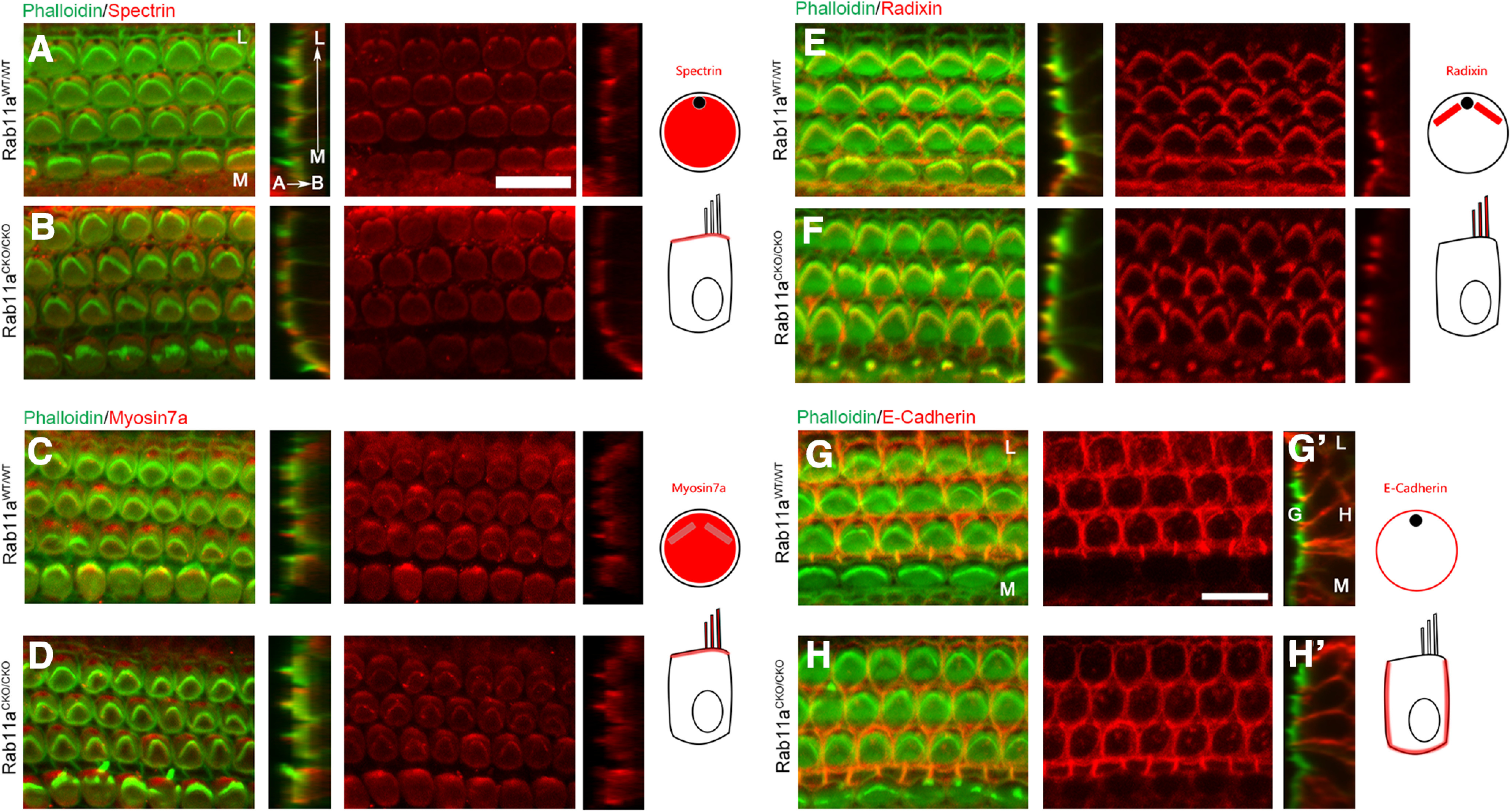
Location of stereocilia architecture components is not altered by loss of Rab11a. ***A–H***, Wild-type (***A***, ***C***, ***E***, ***G***) and Rab11a homozygous conditional knock-out (***B***, ***D***, ***F***, ***H***) cochlea at P2 were stained with phalloidin (stereocilia, green) and β-Spectrin (cuticular plate, red; ***A***, ***B***), MyosinVIIa (hair cell motor protein, red; ***C***, ***D***), Radixin (actin-to-membrane-linking protein, red; ***E***, ***F***) and E-cadherin. All proteins were localized to the apical surface in both WT and Rab11a homozygous samples. Orthogonal views (***A’–H’***) show that normal apical localization is retained for each protein. The schematic diagrams are shown on the right side. Scale bar: 10 μm. L: lateral, M: medial, A: apical, B: basal.

In addition to molecular pathways being located on the apical surface of hair cells, there are apical structures to hair cells that are vital for their physiological function: hearing. Stereocilia are vital for the transmission of sound as they house the mechanoelectrical transduction channels triggered by the bending of the stereocilia. In order for the stereocilia to be formed properly, they must be anchored into the actin meshwork of the cell body, a structure termed the cuticular plate. Although we can see actin at the cuticular plate, we wanted to confirm that other major components were present because Rab11a may take part in the localization of apical proteins and that the absence of important proteins of the cuticular plate will affect HC polarity and function (Y. Liu et al., 2019). Spectrin forms ring structures in the cuticular plate (Y. Liu et al., 2019). In both control and Rab11a CKO cochlear HCs, Spectrin is localized throughout the entire cuticular plate region with the exception of the fonticulous from which the kinocilia emerge (Fig. 5*A*,*B*). We also wanted to confirm that MyosinVIIa was present in the proper localization as it is a motor protein delivering cargo to the stereocilia during building and maintenance and a marker for the cuticular plate (P.Z. Wu and Liberman, 2022). Similarly, in both control and Rab11a CKO cochlear HCs, MyosinVIIa is localized throughout the apical compartment of the cell with a large concentration around the cuticular plate and stereocilia as seen in the orthogonal view (Fig. 5*C*,*D*). Finally, we looked at Radixin expression in the cochlea, which is located near the stereocilia base (Prasad et al., 2020). Radixin links actin bundles in the stereocilia to the adjacent membrane and when ablated results in a similar phenotype where stereocilia bundles are fragmented (Kitajiri et al., 2004). Similar to other proteins analyzed, control and Rab11a CKO HCs had similar localization of Radixin to the stereocilia of HCs (Fig. 5*E*,*F*). These data suggest that Rab11a is not solely responsible for global trafficking of proteins involved in actin organization of stereocilia.

Rab11a has also been found to be responsible for the basolateral localization of E-Cadherin (Desclozeaux et al., 2008), which is expressed only in OHCs. In order to test whether E-cadherin trafficking was altered we compared its expression in controls and Rab11a CKO cochlea. In both, E-Cadherin is expressed at the cell membrane of OHCs (Fig. 5*G*,*H*). Looking at the orthogonal view, we saw clear basolateral expression with no apical expression in either sample (Fig. 5*G’*,*H’*) suggesting that Rab11a does not affect the ability to sort basolateral proteins in the hair cell.

Taken together, these data suggest that loss of Rab11a does not cause a global loss of apical basal sorting ability despite clear role of Rab11 in trafficking in the literature.

### Rab11a interacts with Vangl2 and has certain effect on planar cell polarity

**Figure 6.**
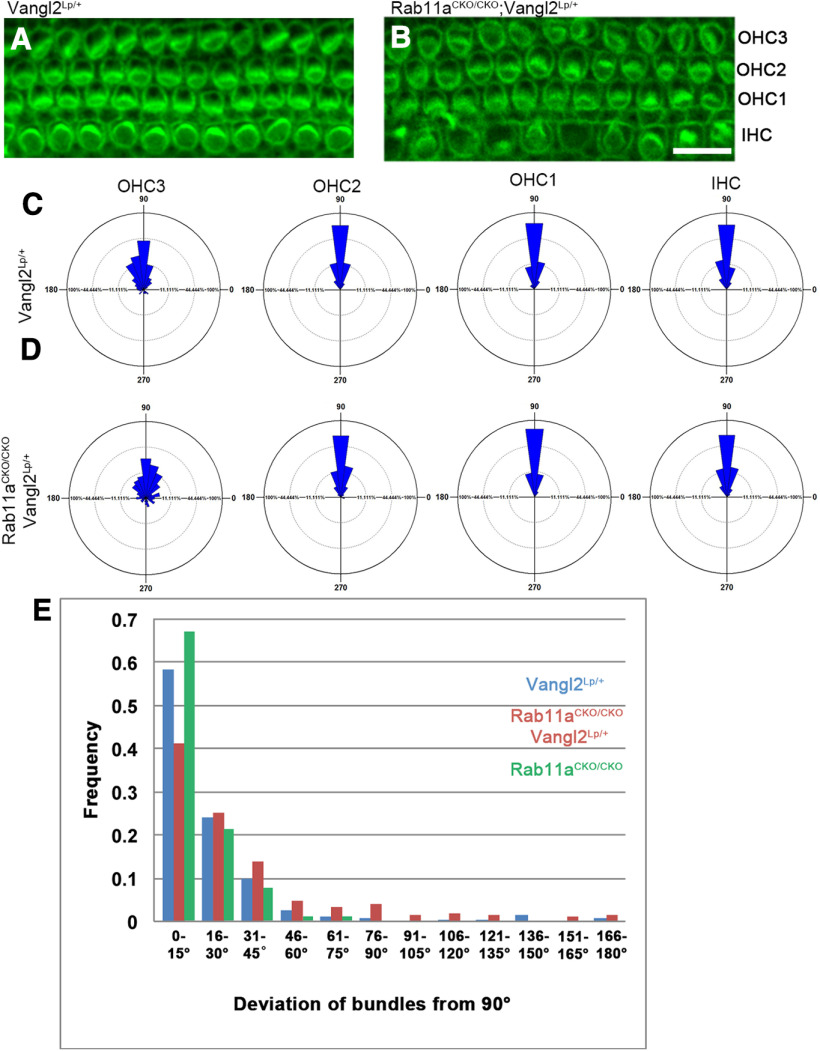
Rab11a regulates hair cell polarity in immature hair cells. ***A***, ***B***, The apical region of the cochlea (P0) in Vangl2-Looptail heterozygote controls (Vangl2Lp/+) and Rab11a homozygous knock-out with Vangl2-Looptail heterozygous (Rab11aCKO/CKO; Vangl2Lp/+) were stained with phalloidin (stereocilia, green) to visualize hair cell polarity along the planar axis. ***C***, ***D***, Oriana graphs showing the distribution of hair cell orientation of Vangl2Lp/+ (***C***) and Rab11aCKO/CKO; Vangl2Lp/+ (***D***). There was a decrease in oriented hair cells in the outermost HC row (OHC3; *p* < 0.0001) and less severely in OHC row 2 (*p* = 0.0204) while other HC row orientation remained unchanged. ***E***, Stereocilia orientation as a function of deviation from 90° was plotted to show the difference in distribution of hair cell orientation among Vangl2Lp/+, Rab11aCKO/CKO;Vangl2Lp/+ and Rab11aCKO/CKO. Scale bar: 10 μm. IHC: inner hair cell, OHC: outer hair cell. A minimum of 300 IHCs and 1200 OHCs were counted per each region of each genotype. Three adjacent fields of view in each region (basal/middle/apex) and five individual animal samples per each condition were included in quantification.

Since the cochlea is a model for PCP with distinct orientation of stereocilia bundles, we sought to determine whether Rab11a affects PCP in the cochlea. We observed that Rab11a does not alter the asymmetric localization of core PCP proteins (Fig. 4). In P0 wild-type cochlea hair cells stereocilia are oriented with their V-shaped or U-shaped stereocilia bundles pointing laterally in a coordinated fashion (Fig. 2*C*). There was no apparent alteration in Rab11a CKO and control cochlea (data not shown). Because many PCP associated protein mutants do not show a severe PCP phenotype, we used a Vangl2-looptail mutant to determine whether Rab11a is involved in any step of the PCP pathway. Vangl2, one of the core components in planar cell polarity (PCP) pathway, is required for correct hair bundle orientation in the inner ear (Montcouquiol et al., 2003). One of the most extensively studied PCP pathway genes knock-out mice is the Looptail mutant (a spontaneous mutation in Vangl2), as it has a stronger PCP phenotype than other Vangl2 alleles. Heterozygotes (Vangl2_Lp/+_) have a weak PCP phenotype that has been extensively used as a sensitized background to detect genes that interact with the PCP pathway (Y. Wang et al., 2006; Macheda et al., 2012; Escobedo et al., 2013); thus, it was used in this study to demonstrate a role for Rab11a in PCP. We weakened the PCP pathway with Vangl2-looptail heterozygous mutant and analyzed the apical section of P0 cochlea where hair cells are not as mature as the basal section. In heterozygous Vangl2-looptail controls, there is slight misorientation in the outermost hair cell (OHC3; Fig. 6*A*,*C*,*E*). In the compound mutant however, there was more dramatic misorientation in the OHC rows 2 and 3 (Fig. 6*B*,*D*,*E*). Quantification of the stereocilia angle revealed a significant increase in the proportion of misoriented hair cells (defined by 30° deviation or more from the mediolateral axis) in OHC3 and a slight increase in OHC2 rows in compound mutants compared with Vangl2-looptail controls (Fig. 6*C*,*D*). However, the IHC row and most medial OHC row had no difference in misorientation. In the statistics of the deviation angle of the direction of the hair bundles of all hair cells, Vangl2-looptail controls showed a similar result with Rab11a^CKO/CKO^, while a higher frequency of larger deviation angle could be seen in compound mutant (Fig. 6*E*). These data suggest that Rab11a has a certain regulatory effect on PCP protein vangl2 but is not playing a leading role, and it may participate in PCP signaling downstream of the core PCP proteins.

### Rab11a regulates ciliogenesis and interacts genetically with IFT88 in the formation of the kinocilia in HCs

**Figure 7.**
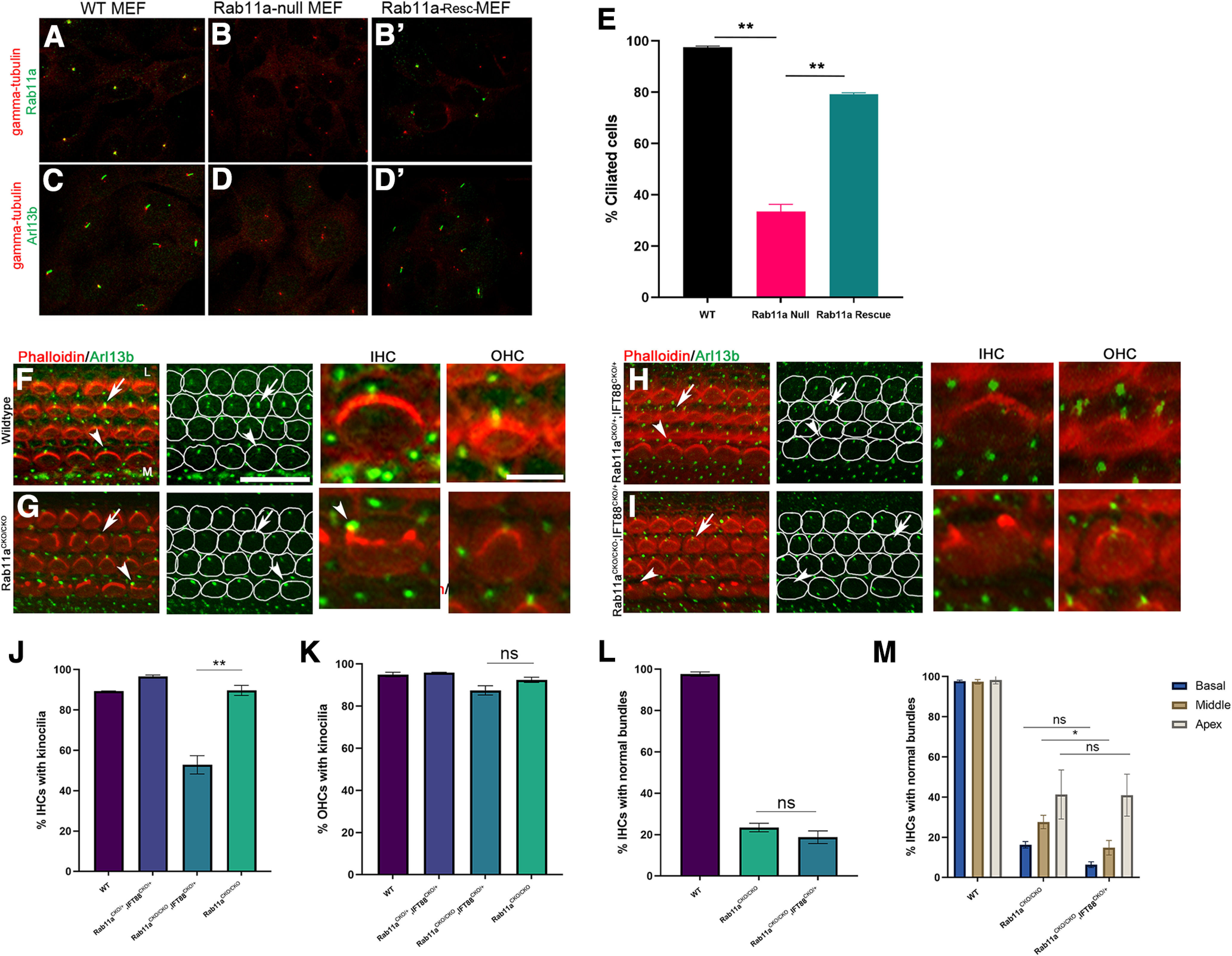
Rab11a plays a role in ciliogenesis and works in concert with IFT88 to form kinocilia. ***A–E***, Wild-type (***A***, ***C***), Rab11a-null (***B***, ***D***) and Rab11a-Resc (***B’***, ***D’***) mouse embryonic fibroblasts (MEFs) were stained with γ-tubulin (basal body, red) and Rab11a (green; ***A***, ***B***, ***B’***) showing the localization of Rab11a to the vicinity of the basal body region in the wild-type (***A***) and Rab11a-Rescued (***B’***) MEFs which is completely lost in Rab11a-null MEFs (***B***). In wild-type (***C***) and Rab11a-Rescued (***D’***) MEFs, ciliation was extensive as shown by the staining with a cilia marker Arl13b (green), while ciliation was lost in Rab11a-null MEFs (***D***). The percentages of cells containing a cilium from wild-type MEFs, Rab11a-null MEFs, or Rab11a-null MEFs transfected with DsRed-Rab11a (Rab11a Rescue) were quantified and plotted with the averages and SEM of three experiments (***E***). Rab11a-null MEFs showed a significant reduction in ciliation which was rescued with Ds-Red-Rab11a transfection. *p*-values equal 0.0013 (WT-Rab11a Null), 0.0026 (Rab11a Null-Rab11a Rescue). ***F–I***, Cochleae from P2 wild-type (***F***), Rab11a homozygous conditional knock-out (Rab11aCKO/CKO; ***G***), Rab11a and IFT88 compounded heterozygous (Rab11aCKO/+; IFT88CKO/+; ***H***), Rab11a homozygous conditional knock-out compounded with heterozygous IFT88 conditional knock-out (Rab11aCKO/CKO; IFT88CKO/+; ***I***) mice were stained with phalloidin (red) and Arl13b (green) to mark the stereocilia and kinocilia, respectively. White outlines of hair cells distinguish hair cell kinocilia from supporting cell cilia. Examples of enlarged views of inner hair cells (IHC) and outer hair cells (OHC) from each respective genotype are shown. Wild-type (***F***) and Rab11aCKO/+; IFT88CKO/+ (***H***) cochlea maintain normal stereocilia bundle morphology with the kinocilium at the vertex of the V-shaped bundle in both inner and outer hair cells. Rab11aCKO/CKO cochlea hair cells have the kinocilia in their inner and outer hair cells despite malformed stereocilia bundles in the outer hair cell (arrow) and inner hair cells (arrowhead; ***G***). The basal body in some inner hair cells is no longer at the lateral edge of the hair cell (arrowhead enlarged IHC; ***G***). Moreover, Rab11aCKO/CKO; IFT88CKO/+ cochlea maintain kinocilia in the outer hair cells (arrow) but while the kinocilium is lost in ∼50% of the inner hair cells (arrowhead; ***I***). M: medial side of the cochlear duct, L: lateral side of the cochlear duct, IHC: inner hair cell, OHC: outer hair cell. Scale bars: 20 μm and 5 μm for the left two and right two columns of images, respectively. ***J***, ***K***, The average and SEM of the percentages of cells containing kinocilia from IHCs (***J***) and outer hair cells (***K***) were quantified and plotted. *p*-values equal 0.0017 indicate differences between Rab11a homozygous conditional knock-out (Rab11aCKO/CKO) and Rab11a homozygous conditional knock-out compound with an IFT88 conditional knock-out allele (Rab11aCKO/CKO; IFT88CKO/+) mutants. The latter has a reduced percentage of kinocilia in IHCs while there is no significant change in outer hair cell kinocilia (***K***). ***L***, Quantification of the morphology of inner hair cell stereocilia bundle of wild-type, Rab11a homozygous knock-outs (Rab11aCKO/CKO) and Rab11aCKO/CKO; IFT88CKO/+ mutant cochleae. The averages and SEM of three samples from each genotype were plotted. *p*-value between Rab11aCKO/CKO and Rab11aCKO/CKO; IFT88CKO/+ is not significant despite a reduction in ciliation in the IHCs of the latter (*p* = 0.3382). ***M***, Quantification of the morphology of inner hair cell stereocilia bundle of wild-type, Rab11a homozygous knock-outs (Rab11aCKO/CKO) and Rab11aCKO/CKO; IFT88CKO/+ mutant cochleae at different regions of the cochlea, the base, middle, and apex. Analysis of the different regions of the cochlea did reveal a reduction in normal stereocilia bundle morphology between Rab11aCKO/CKO and Rab11aCKO/CKO; IFT88CKO/+ that is only significant in the middle region of the cochlea. A two-way ANOVA was used and found a *p*-value of <0.0001 between genotypes and a *p*-value of 0.0013 between regions. Only the middle regions of the homozygous and compound mutant were significantly different. *p < 0.05, **p < 0.001, ***p < 0.001. ns, not significant. A minimum of 300 IHCs and 1200 OHCs were counted per each region of each genotype. Three adjacent fields of view in each region (basal/middle/apex) and five individual animal samples per each condition were included in quantification.

In culture and during *Xenopus* embryogenesis, Rab11 has been shown to regulate cilia development (Knödler et al., 2010; Kim et al., 2012). However, these studies do not distinguish roles of Rab11 family members during ciliogenesis. Thus, we sought to determine the function of Rab11a specifically. First, *in vitro*, we used mouse embryonic fibroblasts (MEFs) isolated from Rab11a-floxed mice in conjunction with a CMV promoter driven retrovirus with Cre allele to create Rab11a-null MEFs (Fig. 7*A–E*; Yu et al., 2014). After inducing ciliation, MEFs without Cre activation (wild-type) express Rab11a at the basal body marked with γ-tubulin (Fig. 7*A*) and had well-formed cilia marked with Arl13b (Fig. 7*C*). In contrast, Rab11a-null MEFs had a loss of Rab11a staining indicated the loss of Rab11a expression (Fig. 7*B*). Additionally, Rab11a-null MEFs have a significant loss of ciliation dropping from 97% ciliation in wild-type to 33% ciliation (Fig. 7*D*,*E*). Ciliation could be rescued by expressing wild-type Ds-Red-Rab11a suggesting that the result is specific to Rab11a (Fig. 7*B’*,*D’*,*E’*).

The cochlea offers a unique opportunity to study the role of Rab11a in ciliogenesis *in vivo*. Sensory hair cells of the cochlea have a precisely oriented kinocilia at the vertex of each stereociliary bundle (Fig. 7*F*). The kinocilia of the cochlea plays a vital role in stereocilia formation. The loss of kinocilia leads to circular stereocilia, which is similar to what we saw in Rab11a CKO hair cells (Fig. 2*B*,*D*; Jones et al., 2008). Based on the observations of Rab11a’s role in ciliogenesis *in vitro* and the observed phenotype of Rab11a CKO cochlea being similar to cilia mutant HCs, we hypothesized that Rab11a may play a role in kinocilia formation. In contrast with our *in vitro* results, Rab11a knock-out in the cochlea did not ablate ciliation in IHCs or OHCs (Fig. 7*G*). Notably, we observed an uncoupling of the kinocilia from the vertex of the stereocilia in a fraction of IHCs (Fig. 7*G*). In the absence of a correctly formed stereocilia bundle, the kinocilia can be observed to the left or right instead of center at the lateral edge (Fig. 7*G*, zoomed IHC).

Because of possibly functional redundancy, like Rab11b or other ciliogenesis components, we used an established cilia mutant to functionally weaken the ciliogenesis pathway in the cochlea. IFT88 is a ciliation protein responsible for transportation within the cilia. Ablation of this protein in the cochlea causes a severe loss of ciliation (Jones et al., 2008). To test the genetic interaction of Rab11a and IFT88 in the ciliogenesis pathway, we first used a double heterozygous conditional knock-out of both Rab11a and IFT88 (Fig. 7*H*). The HCs in these cochleae appeared normal with kinocilia present in both inner and outer HCs. We then analyzed Rab11a homozygous CKO coupled with heterozygous CKO of IFT88 (Fig. 7*I*). These cochleae exhibited a significant decrease in ciliation in the IHC where ∼50% of HCs lost their kinocilia (Fig. 7*I*,*J*). Similar to the Rab11a CKO phenotype, the OHCs were not significantly affected (Fig. 7*I*,*K*). Upon further analysis, ciliation did not present in a clear gradient; ciliation was apparently equal in the apex, middle, and base regions along the cochlear duct in both IHCs and OHCs.

Since the kinocilia has been established in stereocilia formation and patterning, we predicted that adding IFT88 heterozygous conditional knock-out to Rab11a CKO cochlea would cause an increase in the severity of the phenotype seen. Upon quantification of all IHCs along the cochlear duct, there was no clear difference between Rab11a CKO cochlea with or without IFT88 (Fig. 7*L*). However, when separated by region, we could see that the less severe phenotype in the apex was masking the marked differences in the base and middle regions of the cochlea with a 10% decrease in normal stereocilia in the base and a 12% decrease in the middle region (Fig. 7*M*). These data further support the role of kinocilia in stereocilia formation and connects Rab11a’s role in the stereocilia and kinocilia.

Through the use of Rab11a conditional knock-out technology, we were able to demonstrate that Rab11a specifically is necessary for ciliogenesis *in vitro*. In contrast, Rab11a is not necessary for kinocilia formation in the cochlea but may play a role in the orientation of the kinocilia. Further, Rab11a interacts genetically with IFT88 in kinocilia formation giving it a clear role in kinocilia formation. We also noted that compounding heterozygous IFT88 CKO to Rab11a CKO intensifies the stereocilia phenotype in the middle and base IHCs giving further evidence that the kinocilia regulates stereocilia formation.

### Ultrastructure of Rab11a reveals clear stereocilia fragmentation and kinocilia loss in the cochlea

**Figure 8.**
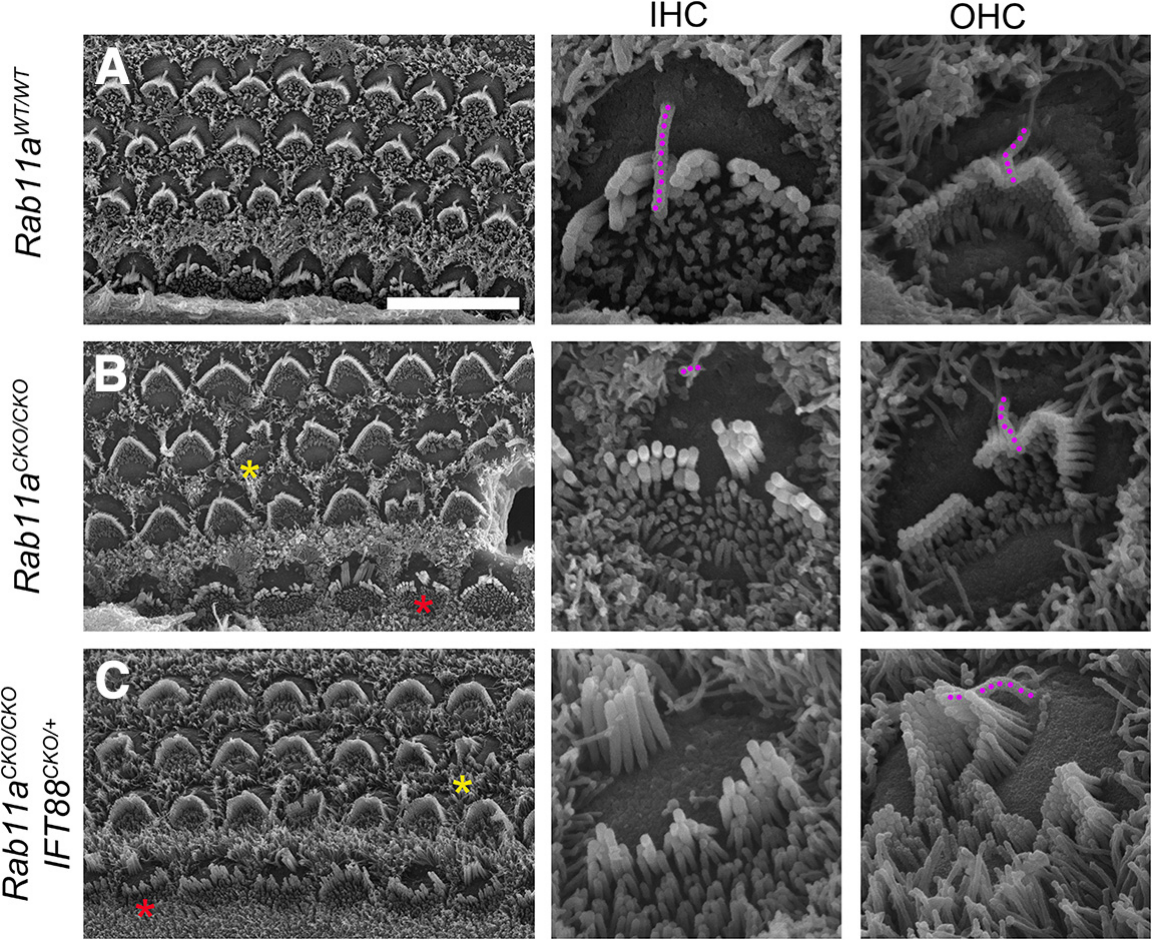
Abnormalities in the hair bundles of Rab11a mutants under scanning electronic microscope. ***A–C***, Scanning electron micrographs (SEM) of wild-type (***A***) Rab11a homozygous Rab11aCKO/CKO (***B***) and Rab11aCKO/CKO; IFT88CKO/+ mutant cochlea (***C***) at P2. The right two columns are IHCs and OHCs at a 4× magnification of the images in the left column. The IHC or OHC shown in the larger magnifications are marked with red and yellow asterisks, respectively. IHC: inner hair cell, OHC: outer hair cell. Scale bar for the left column of images: 10 μm. ***A***, Wild-type cochleae have cohesive inner hair cell (IHC) bundles and outer hair cell (OHC) bundles in the shape of a V with a kinocilium (magenta colored) at the vertex of each stereocilia bundle. They have the characteristic “bare zone” lateral to the stereocilia with microvilli medially. OHC are further along in development and their microvilli are starting to retract. ***B***, Rab11aCKO/CKO IHC stereocilia bundles are not formed properly. They are split (arrow, ***E***) and typically have a kinocilium (magenta colored), which is less discerning because of the change in its length and its mislocalization. Some abnormal OHC bundles were also observed. Kinocilia are present in most cells (magenta colored). The bare zone and microvilli enriched regions lateral and medial to the stereocilia are present. ***C***, Rab11aCKO/CKO; IFT88CKO/+ cochleae display deformed stereocilia and lack the kinocilium in IHCs. Some hair bundles in the outer hair cells are also misshapened and but retain their kinocilia (magenta colored). IHC: inner hair cell, OHC: outer hair cell. Scale bar: 10 μm.

In order to further characterize the phenotypes associated with loss of Rab11a in the cochlea, we turned to scanning electron microscopy (SEM). We analyzed cochlea isolated from P2 mice (Fig. 8*A–C*). Under SEM wild-type cochlea have the characteristic V-and U-shaped stereocilia bundle with the kinocilia at the vertex (Fig. 8*A*). Stereocilia generally couple together based on the lack of space between individual stereocilia. However, SEM reveals that Rab11a CKO HCs experience fragmentation or a loss of adhesion between stereocilia with gaps or missing stereocilia between fragments of bundles in both IHCs and OHCs (Fig. 8*B*). Upon coupling Rab11a CKO with IFT88 heterozygous CKO, it is clear that there is a loss of cilia opposed to a reduction in kinociliary length in IHCs (Fig. 8*C*, IHC). No obvious reduction in kinocilia length or loss of kinocilia in OHCs was noticed (Fig. 8*C*, OHC). In these samples, stereocilia bundles are largely fragmented and present with two sections of stereocilia: one at the lateral part of the cell, and another flatter bundle medially. Additionally, these HCs revealed the partial formation of a circular bundle in the OHC.

Taken together, these data suggest that Rab11a loss causes fragmentation of stereocilia possibly because of a loss of patterning of stereocilia adhesion. Additionally, absent of kinocilia in Rab11a CKO coupled with IFT88 heterozygous CKO suggesting that there is a complete loss in kinociliation opposed to a reduction in kinocilia length.

## Discussion

Until recently, progress in understanding the importance of Rab11a in cellular processes were achieved by using overexpression of dominant negative forms of Rab11a in cell culture (Casanova et al., 1999; Wilcke et al., 2000; Lock and Stow, 2005; Desclozeaux et al., 2008) or knock-down of Rab11a in culture of *in vivo* (Kim et al., 2012; Ossipova et al., 2014, 2015). While these methods have led to many discoveries of the Rab11 family, it is unknown whether the dominant negative constructs also force their dominance over Rab11b or other related proteins because of high amino acid sequence identity. The Rab11 subfamily has three main members: Rab11a and Rab11b sharing a 91% identity differing only at the C-terminus and Rab25 sharing 62% and 61% identity, respectively (Welz et al., 2014). Consequently, many papers report the role of Rab11 instead of Rab11a or Rab11b.

Recently two groups have created knock-out mice where the Rab11a gene is floxed with loxp sites (Sobajima et al., 2014; Yu et al., 2014). Using one of these Rab11a knock-out mice combined with Pax2-Cre to produce knock-out in the inner ear, we show that Rab11a, a protein primarily given the role of apical trafficking and ciliogenesis is vital for formation of the apical processes in the organ of Corti (Ohyama and Groves, 2004; Yu et al., 2014). Rab11a is localized to the basal body region of sensory hair cells and is vital of stereocilia patterning, particularly in IHCs. Additionally, Rab11a plays a clear but nonessential role in kinocilia formation as evident with a loss of ciliation when loss of Rab11a is coupled with deletion of one allele of *IFT88*. To help explain this phenotype, we used a cell system to determine that Rab11a significantly affects ciliation *in vitro*. We demonstrated a genetic interaction between Rab11a and Vangl2 suggesting Rab11a has certain effect on planar cell polarity. Rab11a loss leads to hair cell degeneration and hearing loss. Taken together, these results suggest that Rab11a is involved in several cellular processes more distinct than general apical-basal trafficking and ciliation.

### Rab11a in apical protein localization

This apical trafficking of proteins allows for cellular processes to take place and cellular structures such as actin and microtubule-based projections to form precisely on the cell, while Rab11a was reported to be responsible for apical protein trafficking in epithelial cells (Ullrich et al., 1996; Urbé et al., 1993).

In the organ of Corti, the stereocilia form a distinct and uniform pattern on the apical surface of sensory hair cells. Using antibodies against proteins involved in patterning and formation of the stereocilia, we were unable to detect a change in apical localization of many proteins involved in the patterning of the apical surface including planar cell polarity proteins Vangl2, Frizzled3, and LGN and actin interacting proteins Spectrin, Myosin7a, and Radixin.

These results were surprising considering the phenotype of Rab11a CKO in the gut. Two separate alleles of Rab11a knock-out in intestines resulted in mislocalization of apical proteins (Sobajima et al., 2014; Knowles et al., 2015). Specifically, Ezrin, the form of the ERM protein present in the gut was mislocalized laterally from the normal apical localization(Knowles et al., 2015). Radixin, the ERM protein expressed in the cochlea retained its apical localization in the actin rich cuticular plate and stereocilia. We also analyzed several apically expressed proteins vital for cochlear hair cell development but did not find mislocalization, suggesting that Rab11a is not solely responsible for apical trafficking in the cochlea.

Rab11a has also been implicated in the basolateral localization of E-Cadherin *in vitro* (Lock and Stow, 2005; Desclozeaux et al., 2008) . Like the studies in the intestines and embryo, using Rab11a null mice, E-Cadherin retained basolateral localization in the cochlea on the knock-out of Rab11a (Sobajima et al., 2014; Yu et al., 2014; Knowles et al., 2015; Feng et al., 2017). These data suggest that Rab11a may play a role in E-Cadherin distribution in some systems, but in tissue, there may be redundancies that ensure E-Cadherin is localized appropriately for if it was not, cells would not adhere properly and development of the embryo and organs would be significantly altered.

### Rab11a in planar cell polarity

The role of Rab11 family in planar cell polarity has been implicated in several studies (Ohyama and Groves, 2004; Devenport et al., 2011; Kim et al., 2012; Ossipova et al., 2014, 2015). We analyzed the localization of PCP proteins Vangl2 and Frizzled3 in Rab11a CKO cochlea and found no change in the planar or apical basal localization of these proteins. Additionally, we could not discern a PCP phenotype on knock-out of Rab11a alone. This was not surprising since we believe that other Rab11 family members may be compensating for loss of Rab11a. However, when crossed with the Vangl2-looptail mutant, cochlea did show a planar cell polarity phenotype in the apical region of the cochlea that was most prominent in the most lateral outer hair cell row. There are several possibilities that could account for this phenotype. Developmentally, hair cells in the apical region of the cochlea are less mature than the hair cells at the base. Additionally, more lateral cells are more developmentally immature compared with the medial inner hair cells. This could mean that there is a developmental delay caused by loss of Rab11a. Typically the apex in the cochlea has developed stereocilia by P0 but does not in Rab11a CKO cochlea. The other possibility is that Rab11a helps link the basal body to the machinery that builds the stereocilia. In this model, Rab11a would act as an intermediate in the pericentriolar region between the kinocilia and stereocilia patterning proteins discussed below.

### Rab11a in ciliogenesis in the cochlea

The Rab11 family has been implicated in ciliogenesis through its interactions with Rabin8 that acts as a GEF for Rab8, necessary for membrane extension of the cilia (Nachury et al., 2007; Knödler et al., 2010). In order for ciliation to occur, cilia bound vesicles are sorted at the Golgi for transport to the cilia. Rab11 positive vesicles including Rabin8 destine for the cilia plasma membrane transports these vesicles (Knödler et al., 2010). These data point to a pivotal role for Rab11a in the localization of important ciliation factors to the cilia. However, these studies were done *in vitro* using nonspecific Rab11 knock-down. Here, using a Rab11a knock-out MEF line with complete loss of Rab11a specifically, we definitively show that Rab11a is necessary for ciliogenesis *in vitro*.

*In vivo*, Sobajima et al. (2014) determined that when Rab11a was knocked out in the brain using Nestin driven Cre recombinase, there was no effect on ciliation (Sobajima et al., 2014). Likewise, knock-down of Rab11a in the cochlea did not alter kinocilia formation and presence within the cell. When taken together with the vital role of ciliogenesis in development, it is not surprising that a loss of a single gene does not ablate ciliation. We chose to weaken the ciliation system in the cochlea using knock-out of interflagellar transport protein, *IFT88*. When the ciliation pathway in the cochlea was weakened from heterozygous knock-out, loss of Rab11a did cause nearly 50% loss of ciliation in inner hair cells of the organ of Corti. This suggests that Rab11a does play a vital role in ciliation as IFT88 heterozygous knock-out do not show a loss of ciliation, which may be caused by a partial mislocalization of proteins necessary for ciliogenesis.

### Rab11a in sculpting stereocilia

Recently studies in gut epithelia have revealed that Rab11a is vital for the apical protrusions of the intestinal epithelia, microvilli. With the loss of Rab11a in gut epithelia, microvilli atrophied leading to their reduction in length and width and a loss of apical specificity of microvilli (Sobajima et al., 2014; Knowles et al., 2015; Feng et al., 2017). These data clearly implicate Rab11a in the formation of apical structures. The unique apical structures, the stereocilia of the cochlea are modified microvilli that have lengthened and thickened in a precise formation on the apical surface.

Similar to the gut epithelia, loss of Rab11a in the cochlea causes a malformation in the stereocilia. Unlike reports in the gut, Rab11a stereocilia do not appear blatantly shorter or wider evident by SEM. The defect seen is in the precise patterning of the stereocilia. In late postnatal development, Rab11a loss causes a disruption in stereocilia patterning manifesting as missing or circular stereocilia bundles. In postnatal development, these phenotypes are replaced by stereocilia that are fragmented or missing sections or that are separated into two or more distinct misshapen stereocilia bundles. Our initial hypothesis of the cause of this phenotype was mislocalized Radixin as this protein is responsible for attaching the actin in the stereocilia to the plasma membrane and its close relative Ezrin was mislocalized in the gut (Sobajima et al., 2014; Knowles et al., 2015). Without Radixin there is fragmentation of the stereocilia (Kitajiri et al., 2004). However, there is no difference in Radixin localization or expression in knock-out stereocilia. With this no longer a possibility, we hypothesized that another actin altering protein could be affected which could cause a loss in stereocilia building at specific sites. Since we found no change in actin or β-Spectrin marking the cuticular plate responsible for anchoring stereocilia to the cell, we concluded that this was not the cause for the defect. We noticed that this phenotype resembled the phenotype for Usher proteins, specifically Cadherin23 and Protocadherin15 mutants which lack tip links between their stereocilia leading to a splayed or fragmentation of stereocilia (Michel et al., 2005; Geng et al., 2013). Cadherin23 and Protocadherin15 herterodimerize to form tip links, kinociliary links, and transient lateral links between individual stereocilia for the cohesion of the stereocilia bundle (Goodyear et al., 2005; Longo-Guess et al., 2005; Kazmierczak et al., 2007; Indzhykulian et al., 2013; Cosgrove and Zallocchi, 2014; Maeda et al., 2014; Zhao et al., 2014). Additionally, these mice have profound hearing loss consistent with the Usher protein phenotype and Usher protein’s function as part of the mechanotransduction machinery of the stereocilia. How Rab11a affects tip link requires further study. Also, it remains unclear why there is such a distinguishable difference in the phenotype between IHCs and OHCs early in development.

Recent advances in transcriptomics have highlighted the difference between inner and outer HCs. A variety of papers have recently used microarray or RNAseq to discern the difference between gene expression in IHCs and OHCs (H. Liu et al., 2014; Burns et al., 2015; Li et al., 2016, 2018; Yizhar-Barnea and Avraham, 2017). In addition, mounting evidence suggests that IHC and OHC serve different functions in the cochlea and as a consequence gene expression is different in the cell types (Men et al., 2015). We suspect that Rab11a and Rab11b are expressed at different levels in inner and outer HCs. While immunostaining cannot give an exact quantification of Rab11a in each hair cell type, it appears that the basal body pool of Rab11a is less significant in IHCs compared with OHCs (Fig. 1*C*). However, studies using microarray from adult cochlea found that while Rab11a mRNA was mostly equally expressed in both IHC and OHC, slightly more abundant Rab11a in IHCs (H. Liu et al., 2014). While the cause for the difference in phenotypes between inner and outer hair cells remains unclear, there are examples in the literature where a single mutation can cause different phenotypes in different cell types of the ear. Specifically, a mutation in SorCS2, caused OHC had multiple clusters of stereocilia lacking orientation while IHCs had more stereocilia in a bundle covering a larger surface area while each individual stereocilia was shorter (Forge et al., 2017). This distinctly shows that the same genetic background gives rise to different phenotypes based on the transcriptome of the specific cell. Likewise, this could explain why in Rab11a CKO cochlea IHCs are affected to a larger degree than OHCs.

Taken together, our data indicate that Rab11a plays an essential role in the development of the apical structures of the sensory hair cell. Through differences in inner hair cells and outer hair cells and differences along the basilar membrane, different hair cell types and regions have a stronger or weaker penetrance of the Rab11a CKO phenotype.

## Synthesis

Reviewing Editor: Deanna Smith, University of South Carolina Decisions are customarily a result of the Reviewing Editor and the peer reviewers coming together and discussing their recommendations until a consensus is reached. When revisions are invited, a fact-based synthesis statement explaining their decision and outlining what is needed to prepare a revision will be listed below. The following reviewer(s) agreed to reveal their identity: Artur Indzhykulian.

The reviewers believe that your studies are of potential interest to our readers but feel that substantial revisions are necessary before the paper will be considered for publication in eNeuro. In particular, several sections of the manuscript would benefit from rewriting and proofreading to improve clarity. The introduction needs to be more focused on the experimental work presented. The rationales for specific questions and approaches, and the validity of specific conclusions are not always clear. The reviewers have identified specific weaknesses that are fully described in the critiques below:

Reviewer #1

Hearing critically depends on the correct shaping of hair bundles at the apical surfaces of cochlear hair cells, the auditory sensory cells. This manuscript investigates the mechanisms regulating stereociliary bundle formation between hair cell types in the inner ear, particularly formation of the kinocilium and the stereocilia staircase. Hair bundles are coordinately oriented, displaying unique forms of planar cell polarity (PCP). While coordinated bundle orientation in the sensory epithelium is known to require core PCP protein signaling, the mechanisms linking different levels of polarity remain unclear. The present work shows that a small GTPase protein, Rab11a, is required for proper kinocilia and stereocilia formation. Since they could not detect a PCP phenotype upon knockout of Rab11a in mice, the Rab11a impact on hair bundles does not appear to involve changes in PCP. The topic of the manuscript is potentially of interest and results are generally good. However, there are several issues that reduce enthusiasm for the manuscript in its current form.

Major Concerns

Introduction

“The Introduction requires restructuring to provide a proper rationale that focuses on the experimental work presented. The rationale for the study should be stated more clearly and the background needs better focus. Some of the information included in the discussion would be better placed in the introduction. The authors should include specific research questions that this work addresses, rather than asking readers to infer questions when they reach the results.

“Why did the authors choose to study Rab11a and not other isoforms such as Rab11b? Adding a sentence or two about this in the introduction should clarify their decision-making process. The importance of the connection between PCP and Rab11a for hair bundle formation also needs further discussion.

“Studies mentioned in the introduction are not always relevant, while others that would have been more relevant are not included. For example, much attention is spent on Usher syndrome, while other topics (e.g., Rab11 GTPase proteins) do not receive enough attention. This can cause the reader to lose focus on specific research questions.

“The description of Lim et al., 2011 is confusing. Reword to describe endolytic pathways more clearly - how is this relevant?

“Line 57: All the hair cells within the cochlea are oriented with their “V-shaped stereocilia bundles” pointing to the periphery of the cochlear duct depicting the most distinctive form of planar cell polarity (PCP) in vertebrates should be changed to V- and U-shaped stereocilia” .... And the rest of the manuscript should be checked for this error.

Results

“Many statements in the results are misleading in that they describe work or conclusions not shown in this study. I would urge the authors to edit and proofread the manuscript.

“Line 170: This sentence is confusing: “in the OHCs, more disorganized stereocilia with less severity could been recognized (Figure 1C-F)”. This figure shows IHCs, not OHCs. What is meant by “more disorganized OHC, especially since the data in Figure S2C shows no significant differences?

“Line 271: “Although we can see actin at the cuticular plate, we wanted to confirm that other major components were present” The authors need to include a reference and more description of proteins present in the cuticular plate. They should also explain to why they suspected that Rab11a cKO would impact these proteins.

“Line 283: Toother should be changed to “to other”.

“Line 285: The authors indicate that Radixin is expressed in supporting cells, but this is different from published findings (Prasad et al. 2020 Communication Biology).

“Line 288: It is not clear why N-Cadherin is mentioned because no studies examine N-Cadherin. The authors seem to be looking at E-cadherin localization as a proxy for defects in membrane trafficking, but do not specifically state this.

“Line 306-315: The title of this section is too strong. The Vangl2-looptail mutant needs to be better explained - why does this experiment indicate that Rab11a regulates PCP downstream of PCP proteins? Moreover, the defect in the compound mutant does not look much worse that the defect in the Rab11a cKO alone. Quantitation should be provided.

Discussion

“In general, the discussion is long and some of the information would be better if included in the introduction.

“Line 441: “We demonstrated a genetic interaction between Rab11a and Vangl2 suggesting Rab11a participates in planar cell polarity downstream of core PCP protein localization”. This is a very strong statement and would be more supported if the Vangl2-looptail mutant phenotype had been better described and if the control quantification for the Rab11a cKO was included in Fig 5.

“Line 444-447: Only Myo7a as an Usher protein was used in this study. What cell system has been used here? Most of the studies were done in mice.

Minor concerns

“Fig 1: please use Rab11aCKO/WT and Rab11aCKO/CKO instead of Het and Hom, respectively. Also, labels are a little fuzzy.

“Fig2: Please use the same label for all images. For example, Rab11aWT/WT instead of WT. Part C doesn’t have label and scale bar.

“Fig 3 A and B: it is unclear where Vangl2 and Fz3 proteins are localized, especially since the images are very similar for both. It would be better to use a supporting cell antibody marker like SOX2. Also, Images are sloppy. Please fix all the errors. Part A’ and B’ are unclear and it is hard to understand where these proteins exactly localize.

“Fig 4: Images are sloppy. Please fix all the errors. Also, it would be better to take an image of isolated hair cell to look at the specific localization of each component.

“Fig 6: It would be useful to see an image of Arl13B immunofluorescence after Rab11a rescue. Images are not aligned. Part E: fix the p-values position.

“In Fig7B, kinocilia is missing in IHC of Rab11a CKO mice. I can’t see even at the left or right side of stereocilia. Would it be possible that the Rab11a CKO alone is enough to disrupt kinocilia formation?

Fig 7: Please use the same label for all images. For example, Rab11aWT/WT instead of Rab11a+/+.

“Fig S2: All graphs are small and fuzzy.

“Fig S3: First graph is missing A.

“Line 153: “Inactivation of Rab11a leads to defects in stereocilia bundles of HCs varying along the longitudinal axes and between IHC/OHC of the cochlea”. This sentence is confusing. Please proofread the sentence.

“Line 261: “the planar cell polarity proteins also localize to the apical half of hair cells in controls but on the medial edge in both gentoypes”. The sentence needs proofreading for grammatical and spelling errors.

“Line 291: Investigating if E-cadherin trafficking was altered instead of Rab11a.

“Lines 297-304: The author just repeated the conclusions in a separate paragraph which is unnecessary.

“Line 327: “both IHCs and OHCs are necessary for proper hearing in adult animals” It sounds strange having a sentence like this at the end of result section.

“Line 342-344: Rab11a-null cochlea should be changed to Rab11a null MEFs.

“Line 458: Remove “(discussed below)”.

“Line 600: “our data indicates that Rab11a is plays” remove “is”.

Reviewer #2

The manuscript would benefit from careful proofreading, and some sections of it can be re-written to improve the clarity. Below I provide a list of comments, in no particular order.

Authors do not mention the vestibular phenotype: please consider adding a sentence on whether any obvious vestibular deficit was noticed in these mice.

Authors should use the nomenclature when listing mouse genotypes. It’s not clear whether the WT mice were really wild-types, or just Rab11afl/fl mice without Cre expression. Authors also list Rab11aCKO/CKO mice in figure legends assuming that everyone looking at figures will be reading the manuscript to know which Cre these mice were crossed with. It might be best to list the Cre line as part of the genotype, in agreement with the nomenclature.

Line 55 - please specify that the kinocilium is a true cilium/primary cilium.

Line 81 - I think Goodyear et al., 2010 is one of most convincing studies showing that kinocilial links are at least in part formed by Pcdh15 and Cdh23. Worth referencing. Also, perhaps Indzhykulian et al 2013 showing that transient links are formed by Pcdh15 and Cdh23.

Line 84 - the statement isn’t accurate; please see Webb at al., 2011 showing that isoform-specific Pcdh15-CD2-ko mice have PCP phenotype. Besides, Usher proteins are not the only proteins responsible in forming the bundle (see the latest papers from the Tarchini lab as just one example), and conversely, Usher proteins are not responsible only for bundle - there are some reports that some Usher proteins are localized to the basolateral regions of hair cells near synapses.

Line 108 - its not clear whether this is a speculation at this point, or authors meant to have a reference at the end of this sentence.

Figure 1G-H-I - I suggest authors spend a bit more time formatting the graphs, perhaps assigning unique colors for each genotype, replace “stereocilia” with “bundle” (we can’t see individual stereocilia on these images), and list the statistical analysis used to calculate the p values (I found them in the methods section). The bundles are not clearly visible on images 1A-1D on the left of these graphs, making an impression that the quantification must have been really hard to perform. Were the images scored blindly to the genotype of the animals? Are these confocal images presented as maximum intensity projections? I feel like better quality images can be obtained. Consider using shorter wavelength (405nm) phalloidin for any future work to improve the resolution - I wasn’t able to figure out which one was used in this study.

Line 145: please briefly introduce the Cre line, let the readers know which cell types it is expressed in, and at what age.

Line 182 - did authors mean the degree of deficit?

Figure S2 - please replace ‘stereocilia’ with ‘bundle’ or ‘stereocilia bundle’ as that’s what’s being quantified.

Line 192 - I think the slight difference between apex and base of the cochlea in the knockouts at P2 is less so pronounced as compared to the larger difference between het and homozygote samples. I am not sure whether this finding can be a strong indication that slightly more developed basal hair cells (a couple of days ahead in their development) show a ‘more prominent’ phenotype. If this is an important point why not just look at both cell types a week later?

Line 199 - ‘determine hair cell structure’ - please clarify

Fig 2C - the blue overlaid text is hard to read, consider changing. Also consider adding the age of the animals next to the genotype. Fig 2B is listed as knockout. Is panel C also from a knockout mouse? While authors mention missing hair cells on this panel, it’s hard to tell if that’s the case since the blue channel isn’t showing well. Consider increasing contrast or changing the color to magenta.

Line 207: it’s not clear how authors conclude that ‘the hair cells without stereocilia were lost by 4 weeks’. Was there quantification done for hair cells (myosin positive) without stereocilia at earlier ages vs. the same at older age? All one can conclude from panel 2C is that there are missing hair cells in the cochlea at that age.

Line 208 - not sure what “abnormal connections” refers to. Please clarify what connections are being discussed here. Of note, the IHC bundles are hard to preserve for SEM imaging, and they often look disorganized as a result of dissection artifacts even in normal cochlear samples. It would be important for authors to confirm the few bundles they show on these SEMs are truly representative of the entire cochlea. I would recommend adding lower magnification images that would include 15-20 hair cell bundles (IHCs and OHCs) as SEM image for each genotype.

Fig 2H: again, is WT really a WT mouse? Does the ‘hom’ refer to the Rab11aCKO/CKO that is PAX-Cre+? there is plenty of space there, please list the proper genotypes. What frequencies were used for ABR testing? The methods section first mentions “8,16,24,32 kHz” then says that “Amplitudes (mV) and latencies (ms) of the initial ABR peak 746 (wave I) were then determined at frequencies 11.3, 16 and 22.6 kHz”

Line 213: “These data indicate that Rab11a is responsible for the maintenance of hearing:” it’s hard to agree with this statement, as authors don’t show normal hearing in these mice at any time during development, so how can one argue that Rab11a is responsible only for maintenance, and not at least in some way, for development of normal hearing? Please revise.

Line 229: were any of these antibodies validated? Please make sure to discuss this, perhaps in the methods section.

Lines 234, 250: change to ‘these data suggest’

Line 283: ‘toother’ replace with ‘to other’

Line 314: please reference the mutant that’s being discussed in the sentence, explain its significance.

Fig 5A-B: what is the age of the animals? Make sure to list it in figure legends in addition to the main text.

Line 345: please include an example image of Rab11a rescue following introduction of DsRed-Rab11a in Fig 6A-E. Also, it’s not clear to me how authors determined whether the cell is ciliated or not. All panels seem to have gamma-tubulin signal that might be a primary cilium, right? The methods sections reads “Cells were scored for ciliation and analyzed as described below.” But perhaps I missed where exactly, as there was nothing below.

Figure S3 - label “A” is missing

Line 408: authors say ‘stereocilia are fragmented’ ... I think they mean bundles are fragmented.

Lline 416: is the loss of kinocilia go alongside with the loss of basal body?

Line 534: I would refrain from speculating outside the results presenting in this paper - there is no evidence for Rab11b to compensate for Rab11a. It is not clear to this reviewer how the loss of apical accumulation of Rab11b in Rab11a-ko mice supports this statement. Please clarify or remove.

Line 569: again, there is no evidence that Rab11a regulates trafficking of tip link proteins. Authors could check the immunolabeling for these proteins or perform more rigorous SEM or TEM microscopy to see whether any of stereocilia links are missing. If these mice were missing tip links, their hearing loss would have been more profound, and not just a 40dB threshold shift. Please add more supporting evidence or remove this speculation, as I think it’s far-reaching.
